# Distinct inflammatory marker profiles of adolescents and young adults among the HIV risk, infection, and treatment spectrum

**DOI:** 10.1038/s41467-026-78034-6

**Published:** 2026-09-25

**Authors:** Huan Vinh Dong, Gielenny M. Salem, Tara Kerin, Kwan Hou Tang, Ruth Cortado, Myung Shin-Sim, Elizabeth Mayfield Arnold, Dallas Swendeman, Nicole H. Tobin, Anne W. Rimoin, Grace M. Aldrovandi, Otto O. Yang, Yvonne J. Bryson, Suan-Sin Foo, Karin Nielsen-Saines

**Affiliations:** 1 Department of Pediatrics, Division of Pediatric Infectious Diseases, David Geffen School of Medicine, University of California, Los Angeles, CA USA; 2 A*STAR Infectious Diseases Labs, Agency for Science, Technology and Research (A*STAR), Singapore, Singapore; 3 Department of Microbial Sciences in Health, Cleveland Clinic Research, Cleveland Clinic, Cleveland, OH USA; 4 Department of Medicine, Statistics Core, David Geffen School of Medicine, University of California, Los Angeles, CA USA; 5 Department of Psychiatry, University of Kentucky College of Medicine, Lexington, KY USA; 6 Department of Psychiatry & Biobehavioral Sciences, David Geffen School of Medicine, University of California, Los Angeles, CA USA; 7 Department of Epidemiology, UCLA Jonathan and Karin Fielding School of Public Health, Los Angeles, CA USA; 8 Department of Medicine, Division of Infectious Diseases, David Geffen School of Medicine, University of California, Los Angeles, CA USA

**Keywords:** HIV infections, Chemokines, Prognostic markers, Immunopathogenesis

## Abstract

Youth living with or at HIV-risk exhibit distinct immune perturbations during HIV exposure, infection, and antiretroviral therapy (ART). Understanding immune perturbations is critical for development of tailored prevention and enhanced therapeutic strategies. We performed plasma proteomic profiling of 49 biomarkers using Luminex multiplex assay in 262 participants (mean age of 21.9 years), including healthy controls, high-risk youth without HIV (HRY), newly diagnosed youth living with HIV (NY), and previously diagnosed youth on ART for over 24 months (PY), and report distinct immune landscapes. Among HRY, we observe dysregulation of circulating interferon-driven inflammation. NY display overt systemic inflammation at diagnosis, which partially resolves but does not fully normalize within 24 months of ART. PY display largely normalized immune profiles, though circulating signatures consistent with residual checkpoint activation persist. Our results identify conserved and stage-specific, circulating inflammatory modulators and key transcriptional regulators delineating HIV susceptibility, progression, and long-term treatment responses.

## Abstract (web summary)

Nielsen-Saines and colleagues identify unique and divergent immune perturbations across groups of youth living with or at risk of HIV. Their findings shed light on conserved and stage-specific that may underlie variations in outcomes and treatment success for HIV.

## Introduction

Human Immunodeficiency Virus (HIV) infection remains a major global health burden, with approximately 1.3 million new infections reported annually worldwide^1^. A substantial portion of these occur among adolescents and young adults, a population uniquely vulnerable due to intersecting immunological and behavioral factors^2,3^. Youth living with HIV (YLWH, early/late adolescents under age 25 years) represent a critical demographic in the HIV epidemic, as their immune systems are still maturing, and their risk behaviors, such as substance use, inconsistent access to care, and exposure to sexually transmitted infections (STIs), can influence both acquisition and disease progression^3,4^. In the United States, youth at substantial risk for HIV acquisition should receive routine HIV pre- and post-exposure prophylaxis^5^.

HIV infection is associated with systemic immune activation and despite the widespread availability and transformative impact of combination antiretroviral therapy (cART) in converting HIV from a fatal illness to a manageable chronic disease, individuals on cART continue to exhibit persistent immune dysregulation^6–11^. This residual activation is associated with chronic inflammation, increased soluble immune checkpoint markers, and increased susceptibility to opportunistic infections and non-acquired immunodeficiency syndrome (non-AIDS) comorbidities, including cardiovascular and neurocognitive disorders^8,12,13^. Nevertheless, studies in adults have suggested that early potent cART during acute HIV infection may reduce viral reservoir size, disrupt residual replication, mitigate chronic immune activation/ dysregulation and facilitate better long-term HIV control ^14–16^.

The adolescent immune system is inherently different from that of adults, in that there is still some thymic function, which potentially would render young individuals more responsive to HIV viral eradication attempts following early treatment. Emerging data suggest that early therapy during acute HIV infection in both adults and children quantitatively modifies HIV persistence and HIV-specific immune responses and may enable strategies for HIV remission^17–19^. Youths represent an important population in HIV epidemiology, with 13–24 years old accounting for 19% of new infections in the United States^20^. As this population ages, they are at risk for developing non-AIDS related conditions^21^, however, data are lacking regarding the specifics of their increased inflammation and immune dysregulation. Adolescents tend to have less knowledge regarding HIV, less condom utilization, and more substance use than adults, which can increase HIV acquisition risk. Furthermore, racial/ethnic and sexual minority youth also have a higher incidence of infection, with male-to-male sexual contact accounting for 67% of new infections in 2022 in the U.S ^20^.

The Adolescent Trials Network (ATN) Comprehensive Adolescent Recruitment and Engagement Strategies (CARES) studies were multifaceted, linked studies that evaluated distinct youth populations at risk of HIV infection (ATN 149), YLWH who were identified since the time of or following acute infection (ATN 147), and youth with established HIV diagnosis at enrollment (ATN 148)^22–25^. ATN 148^24^ and 149^22^ were randomized clinical trials, whereas ATN 147 was a cohort study of newly diagnosed youths initiating cART on or after the period of acute infection^23^. ATN 148 evaluated specific interventions focused on improving viral load adherence in participants with established HIV infection who did not have virologic suppression at the time of enrollment, although the study also enrolled a subset of participants with undetectable viremia^24^. ATN 149 was a randomized clinical trial of youths at high-risk for HIV infection, which evaluated 3 distinct strategies for optimization of HIV prevention^22^. The population of youth enrolled in ATN studies was representative of adolescents and young adults living with HIV in the U.S^25^. This cohort of youths provides the opportunity to evaluate the trajectory of immune activation in young adults in distinct phases of viral interaction. That is, before HIV infection, following recent or acute HIV infection (prior to antiretroviral initiation and at 12 and 24 months post treatment), and those with established HIV infection, as compared to control individuals from a similar age group recruited from non-HIV studies.

In the present study, we evaluated whether young individuals below 25 years of age have distinct developmental changes to their immune systems, with HRY harboring pre-existing immune alterations that may increase vulnerability to infection^3^. Similarly, longitudinal immune profiling in newly diagnosed youth (NY) provides a crucial understanding of the trajectory of immune recovery and the persistence of inflammation following ART initiation. Notably, youth with longer-term established HIV infection may also display distinct immune signatures, underscoring the importance of age-specific investigations into HIV immunopathogenesis^26,27^. Plasma proteomics enables high-resolution profiling of systemic immune mediators, capturing circulating cytokine networks, immune regulatory pathways, and inflammation-related processes that serve as indicators of systemic immune dynamics^28^. By integrating multiplex cytokine measurements with viral load data and exploratory pathway enrichment analyses, we aimed to generate novel hypotheses regarding candidate host immune responses prior to and during HIV acquisition, progression, and treatment.

## Results

Table 1 provides details of participants enrolled in the three ATN studies and controls. In total, 262 participants were evaluated, 93 enrolled from ATN 147 at baseline, and 63 and 56 were evaluated at 12 and 24 months, respectively, with an undetectable viral load on both follow-up time points. ATN 147 participants are referred to in the figures as NY (Newly-diagnosed Youth), ATN 148 participants as PY (Previously-diagnosed Youth), and ATN 149 HRY (High-Risk Youth). Age was comparable across all groups, although the healthy control group had a slightly higher mean age (25 years), with a statistically significant difference noted between groups. The youngest participant in the present analysis was 16 years old at enrollment, and the oldest was 24 years old per the inclusion criteria. In the healthy controls, the youngest participant was 20 years of age and the oldest was 27 years. All participants were male. The ATN study demographics, particularly those in the NY/ ATN 147 and HRY/ ATN 149 groups, matched the race/ethnicity parameters reported by the CDC for youth living and at risk for HIV, being predominantly Black and Hispanic individuals^20^. High rates of STIs were reported for all ATN participants, averaging 10% for gonorrhea, 8% for chlamydia, and 20% for syphilis in all ATN groups (Table 1). Additional details on visit-level STI occurrence, including the number of unique participants with at least one STI and the distribution of concurrent infections, are summarized in Supplementary Table 1.

**Table 1:** Demographic and clinical characteristics of study participants enrolled in the three ATN studies and Healthy controls

	All (*n* = 262)		NY 0M (*n* = 93)		PY (*n* = 24)		HRY (*n* = 110)		Controls (*n* = 35)		*P* value
**Age**	Mean	SD	Mean	SD	Mean	SD	Mean	SD	Mean	SD	
	21.9	2.4	20.9	2.23	22.4	1.71	21.7	1.94	25.1	1.9	<0.001
**Race/Ethnicity**	*n*	%	*n*	%	*n*	%	*n*	%	*n*	%	
Asian/Native American/Alaska Native/Pacific Islander/Other	21	8.0%	7	7.5%	1	4.2%	11	10.0%	2	5.7%	<0.001
Black	87	33.2%	54	58.1%	7	29.2%	24	21.8%	2	5.7%	
Hispanic	104	39.7%	25	26.9%	11	45.8%	58	52.7%	10	28.6%	
White	50	19.1%	7	7.5%	5	20.8%	17	15.5%	21	60.0%	
**STIs at blood draw**	*n*	%	*n*	%	*n*	%	*n*	%	*n*	%	
Gonorrhea	22	8.4%	14	15.1%	1	4.2%	7	6.4%	0	–	0.07
Chlamydia	18	6.9%	10	10.8%	1	4.2%	7	6.4%	0	–	0.4
Syphilis	45	17.2%	22	23.7%	10	41.7%	13	11.8%	0	–	0.002

### Distinct immune signatures in high-risk youth and youth living with HIV

**Fig. 1:**
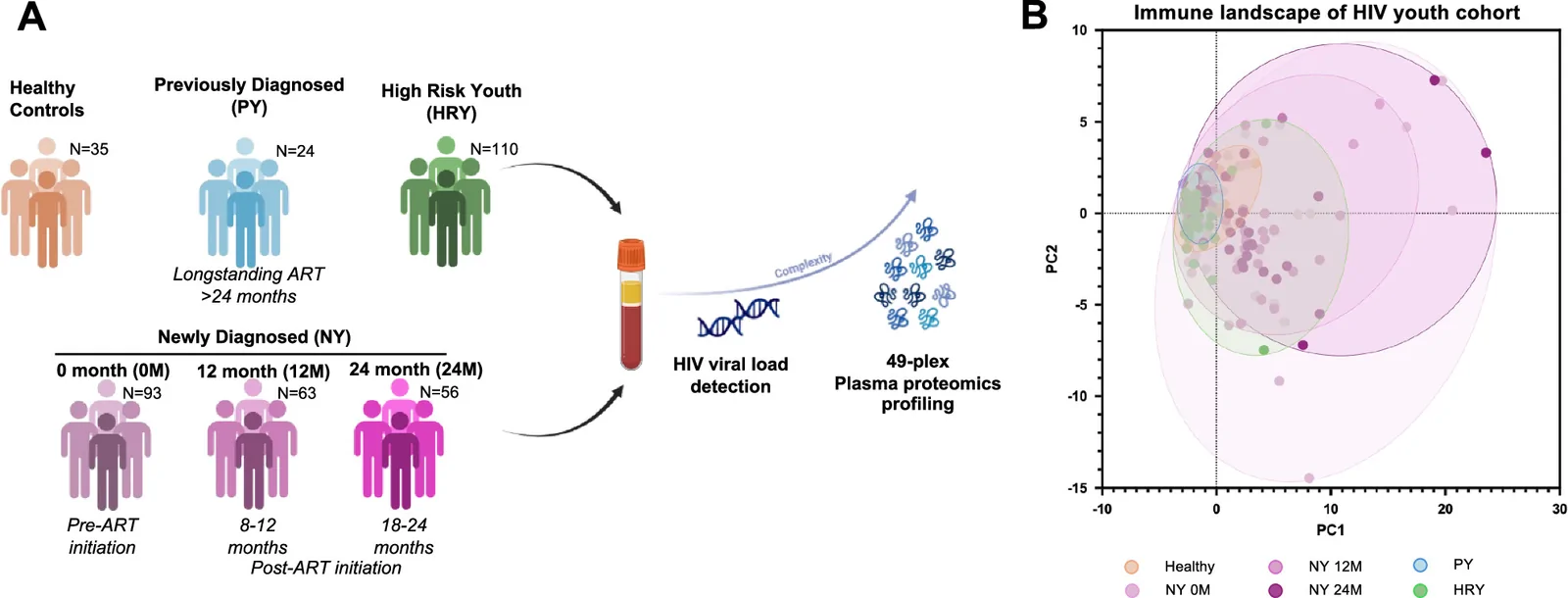
Schematic representation of HIV youth cohort. **A** Schematic representation of the study cohorts, comprising healthy controls (*n* = 35) and youth at risk of or living with HIV (*n* = 227). HIV-exposed participants were stratified into: (i) previously diagnosed youth (PY, *n* = 24), on ART for >24 months (long-term treated); (ii) high-risk youth (HRY, *n* = 110), without HIV infection but at elevated risk of disease contraction; and (iii) newly diagnosed youth (NY, *n* = 93) followed over time. NY were followed longitudinally at three time points relative to antiretroviral therapy (ART) initiation: baseline or prior to ART (0 M, *n* = 93), 12 months (12 M, *n* = 63), and 24 months (24 M, *n* = 56). HIV viral load was quantified by qRT-PCR, and plasma proteomes were profiled using multiplex proteomics. HIV viral load was measured by qRT-PCR, and plasma proteomes were profiled using multiplex proteomics. **B** Principal component analysis (PCA) of plasma proteomes showing the immune landscape of the HIV youth cohort. Each point represents an individual sample, color-coded by group: Healthy, NY-0M, NY-12M, NY-24M, PY, and HRY. HIV Human immunodeficiency virus, qRT-PCR Quantitative reverse transcriptase- polymerase chain reaction. The illustration generated in (**A**) is original and was in BioRender. Salem, G. (2026) https://BioRender.com/zzaqddb.

**Fig. 2:**
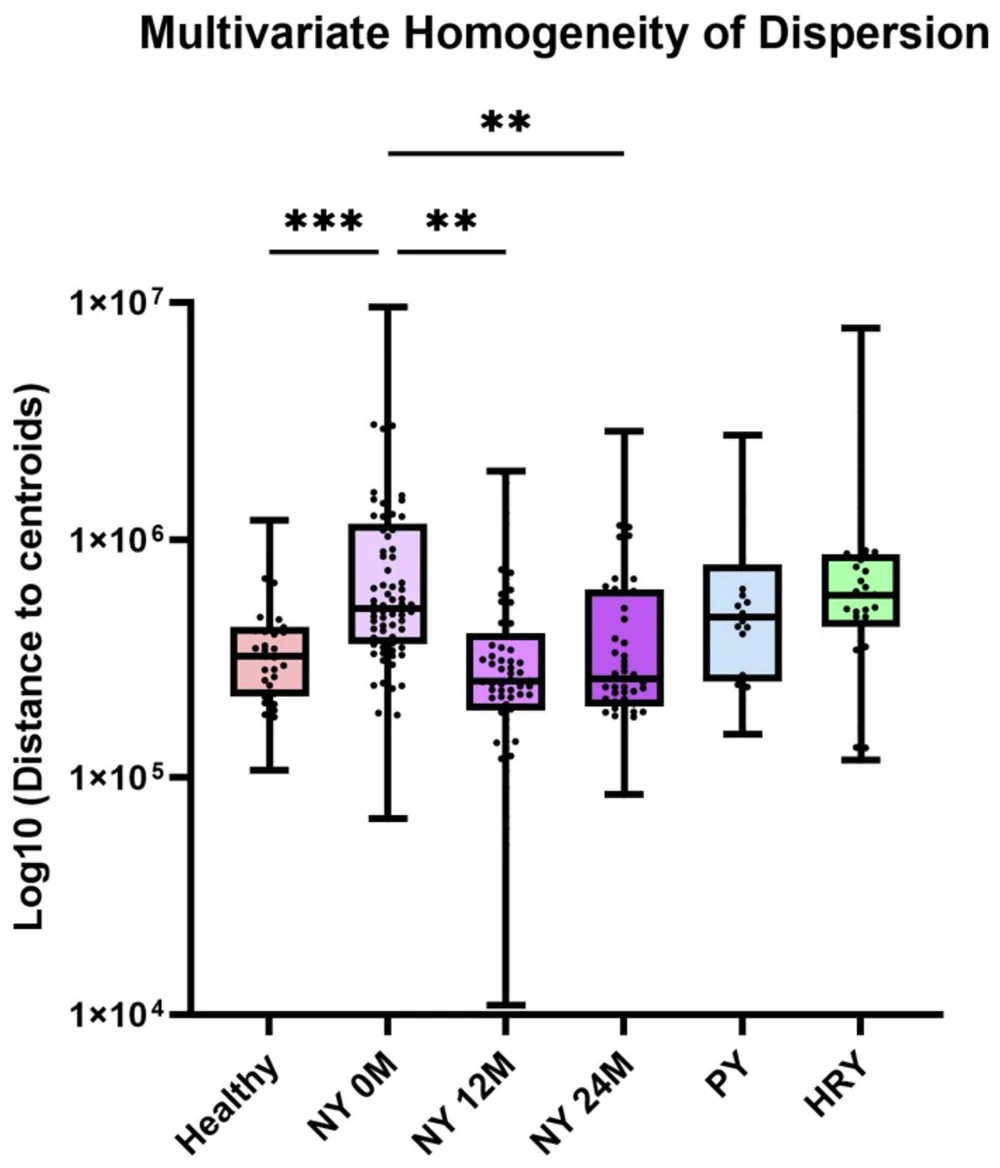
Multivariate homogeneity of dispersion (PERMDISP) analysis across study cohorts. Distance-to-centroid values were evaluated across health controls (*n* = 35), newly-diagnosed youth at three time points relative to antiretroviral therapy (ART) initiation of baseline or prior to ART (0 M, *n* = 87), 12 months (12 M, *n* = 61), and 24 months (24 M, *n* = 50), previously diagnosed youth (PY, *n* = 20), and high-risk youth (HRY, *n* = 33), without HIV infection but at elevated risk of disease contraction. Statistical analysis was made via a one-way Welch’s ANOVA (*P* = 0.0003) with a post-hoc Dunnett’s T3 test for pairwise comparisons. Box-and-whiskers plots display all points, center lines indicate medians, the 25th and 75th interquartile ranges (IQR), and whiskers indicate the minimum to the maximum values. **P < 0.01, ***P < 0.001.

To characterize systemic circulating immune profiles associated with HIV risk, acquisition, and treatment, we profiled plasma proteomes from a clinically characterized cohort of 262 adolescents and young adults, stratified into healthy controls (*n* = 35), high-risk youth (HRY, *n* = 110), newly diagnosed YLWH (NY, *n* = 93), and previously diagnosed youth on long-term ART (PY, *n* = 24) (Fig. 1a). NY participants were sampled longitudinally at three time points: baseline or prior to ART initiation (0 months, 0 M; *n* = 93), at 12 (12 M, *n* = 63) and 24 months (24 M, *n* = 56) post-ART. Principal component analysis (PCA) of plasma immune signatures revealed near-overlapping group-level clusters for most groups (Fig. 1b). NY participants displayed the most widespread dispersion for all time points (0, 12, 24 M), while overlapping with all other groups including healthy control, PY, and HRY groups (Fig. 2), indicating a greater heterogeneity in the circulating immune profiles during early HIV infection and following ART initiation. In contrast, PY participants on long-term ART showed immune profiles that tightly overlapped with healthy controls, suggestive of a shift toward protein profiles resembling healthy baseline states. Interestingly, HRY displayed broader clustering with partial overlap with the PY group.

### Immune dysregulation in high-risk youth mirrors microbial activation

**Fig. 3:**
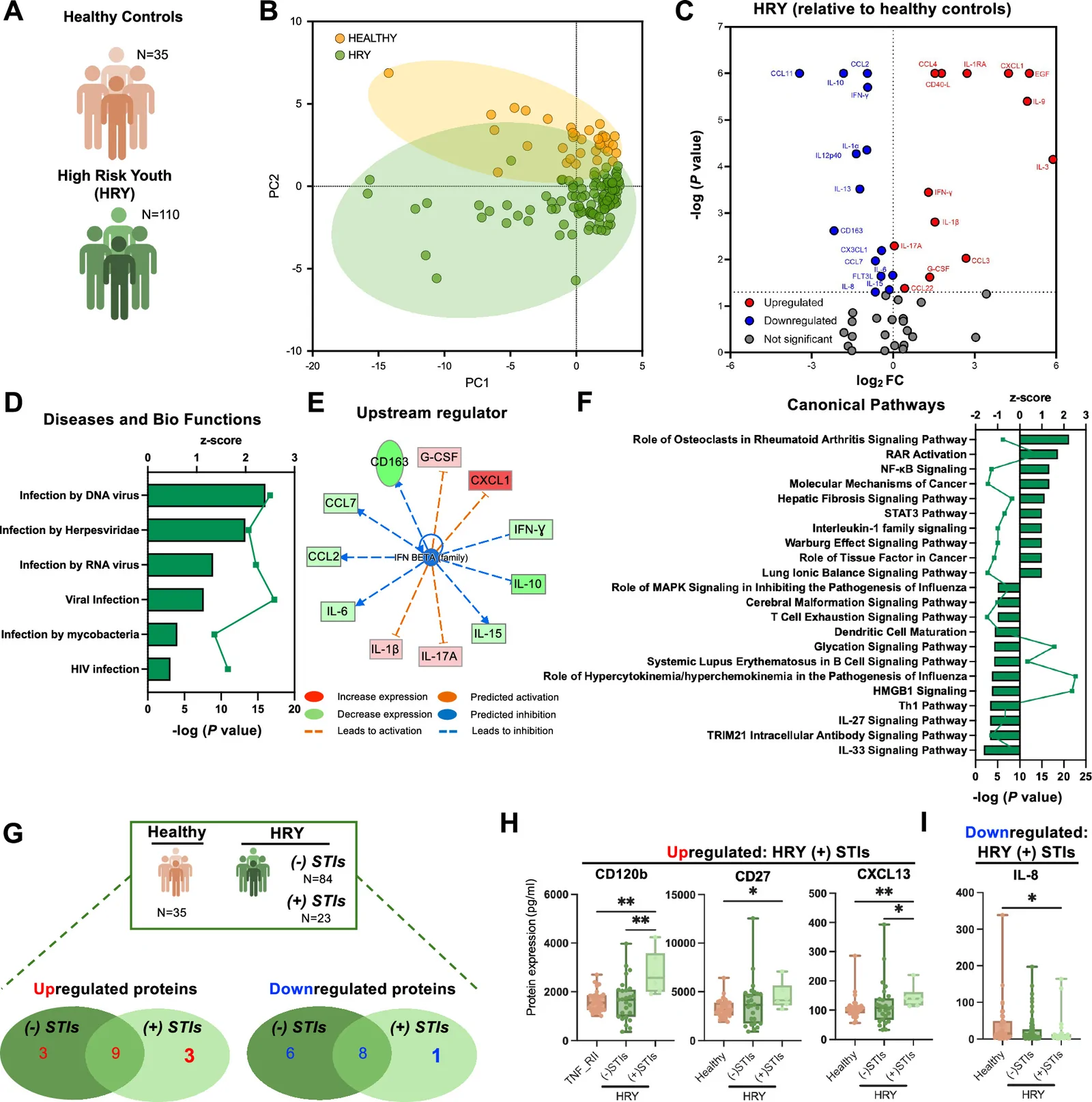
High-risk youth exhibit a distinct immune landscape which mimics immune activation patterns associated with microbial exposure. **A** Schematic of healthy (*n* = 35) and high-risk youth (HRY, *n* = 110) cohorts. **B** Principal component analysis (PCA) of plasma proteomes from healthy (yellow) and HRY (green) individuals; dots represent independent samples. **C** Volcano plot of differentially expressed plasma proteins in HRY compared to healthy controls (two-tailed Mann–Whitney test). Significantly upregulated (red) and downregulated (blue) proteins meet the threshold of *P* < 0.05 and log_2_ fold-change (FC) > 1.1 or −1.1 (horizontal dotted line indicates *P* value cut-off). **D** Diseases, biological functions, and physiological processes associated with microbial infections in the HRY cohort. **E** Graphical summary of transcription factors regulating the expression of IFN-β family; **F** Canonical pathway enrichment (−log(*P* value), bars) and predicted activation state (*z*-score, overlaid line) in HRY (right-tailed Fisher’s exact test (*P* < 0.05). **G** Schematic representation of overlap of differentially expressed proteins in HRY without (−STIs) and with (+STIs) sexually transmitted infections relative to healthy controls, as shown separately for upregulated and downregulated proteins. **H**, **I** Plasma protein levels (pg/ml) of upregulated (**H** CD120b (Healthy vs +STIs = 0.0020; −STIs vs +STIs = 0.0031); CD27 (Healthy vs +STIs = 0.00296); and CXCL13 (Healthy vs +STIs = 0.0080; −STIs vs +STIs = 0.0208) and downregulated (**I** IL-8 (Healthy vs +STIs = 0.0473) proteins across healthy (*n* = 35), −STIs (*n* = 84 for (**I**), *n* = 26 for (**H**)), and +STIs (*n* = 23 for (**I**), *n* = 6 for (**H**)) cohorts. Ce*n*ter lines i*n*dicate medians, box boundaries indicate 25th and 75th interquartile ranges (IQR); whiskers show the range; individual data points are overlaid. Statistical significance was determined using Kruskal–Wallis test with two-sided post-hoc Dunn’s multiple comparisons test (*P < 0.05, **P < 0.01). ADAM11 ADAM metallopeptidase domain 11, CCL-2 C–C motif chemokine ligand 2, CD40LG CD40 ligand, CXCL1 C-X-C motif chemokine ligand 1, EGF epidermal growth factor, IL-6 interleukin 6, G-CSF granulocyte colony-stimulating factor, CD163 cluster of differentiation 163, IFN-γ interferon gamma. The illustrations generated in (**A**) and (**G**) are original and were created in BioRender. Salem, *G.* (2026) https://BioRender.com/4ghyq72.

We next characterized the systemic immune profiles of HRY relative to healthy controls. Despite the absence of HIV infection, HRY individuals exhibited marked alterations in systemic circulating protein signaling (Fig. 3a, b). Differential expression analysis identified 82 significantly dysregulated plasma proteins (adjusted *P* < 0.05, |log₂FC| > 1.1), defined by the upregulation of 13 pro-inflammatory mediators and the downregulation of several innate immune effectors (Fig. 3c). Notably, among the upregulated markers were key β-chemokines, such as C–C motif chemokine ligand 3 (CCL3) and CCL4, known as HIV suppressive factors by acting as natural ligands for the CCR5 co-receptor^29,30^. Gene ontology (GO) enrichment analysis linked these proteins to pathways associated with microbial activation, including Toll-like receptor signaling, cytokine-cytokine receptor interactions, and chemokine signaling (Fig. 3d).

Upstream transcriptional regulator analysis further revealed a complex inflammatory network. Specifically, we observed the predicted inhibition of type I interferon-associated signaling and regulatory nodes, including cluster of differentiation 163 (CD163), CCL2, CCL7, and interleukin (IL)-6, IL-10, and IL-15. This occurred alongside the predicted activation of pro-inflammatory mediators (granulocyte colony-stimulating factor, G-CSF; C-X-C motif chemokine ligand 1, CXCL1; IL-1β, and IL-17A), suggesting a dysregulated circulating interferon-driven inflammatory network within the high-risk cohort (Fig. 3e). Mapping these proteins onto canonical immune pathways was highly consistent with a systemic environment mimicking acute microbial challenge, with predicted activation of NF-κB, signal transducer and activator of transcription 3 (STAT3), and IL-1 signaling cascades (Fig. 3f). Notably, the enrichment of the “pathogenesis of influenza” pathway suggests a hyper-inflammatory state in HRY despite the absence of viral infection. This was further characterized by the predicted inhibition of IL-33 signaling and pathways mapping to checkpoint regulation, indicating a shift from homeostatic regulation toward potentially maladaptive immune signatures.

**Fig. 4:**
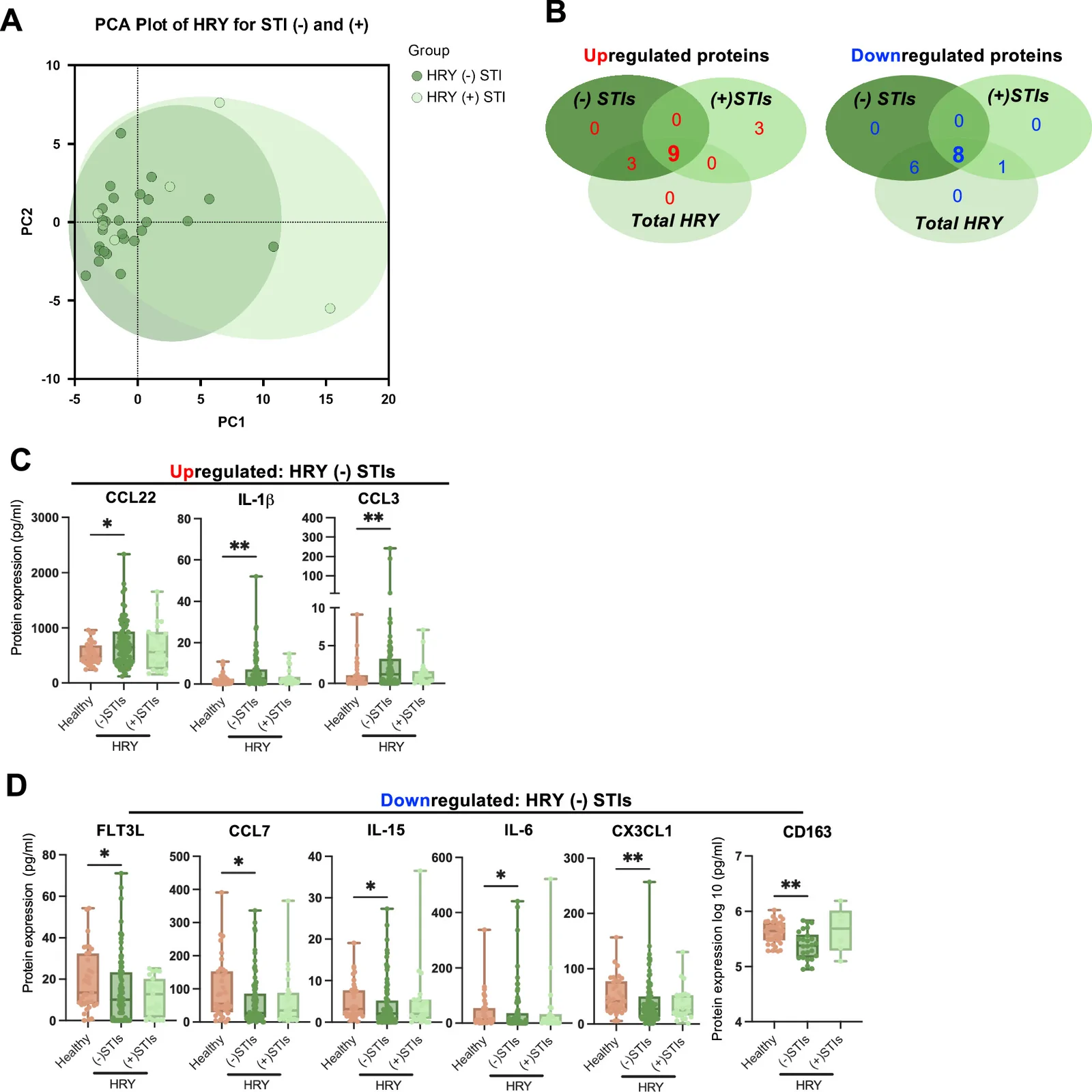
Stratified analysis of high-risk youth (HRY) immune profiles based on sexually transmitted infection (STI) status. **A** Principal component analysis (PCA) plot of plasma proteomes showing overlapping immune profiles of HRY patients who are STI-negative (dark green, *n* = 84) and STI-positive (light green, *n* = 23); each point represents one sample. **B** Venn diagram of significantly upregulated and downregulated inflammatory markers shared between HRY individuals who are STI-negative only, STI-positive only, and combined STI-negative/positive groups, relative to healthy controls. **C**, **D** Protein expression profiles (pg/ml) of plasma immune mediators identified to be uniquely upregulated (**C**) or downregulated (**D**) in STI-negative individuals (*n* = 84) among HRY individuals, relative to healthy controls. Data in (**C**) and (**D**) are represented as boxplots with individual data points; center lines show the medians, boxes indicate the 25th and 75th interquartile ranges (IQR), and whiskers extend to the minimum and maximum values. Statistical significance was determined using Kruskal–Wallis test, with a two-sided post-hoc Dunn’s multiple comparisons test; *P values are indicated (*P < 0.05,**P < 0.01). CCL3 C–C motif chemokine ligand 3, CD163 cluster of differentiation 163, FLT3L Fms-related tyrosine kinase 3 ligand, CCL7 C–C motif chemokine ligand 7, IL-15 interleukin 15, IL-6 interleukin 6, CX3CL1 C-X3-C motif chemokine ligand 1.

To determine the influence of co-infections, we stratified the HRY cohort by STI status. This revealed largely overlapping immune signatures; the majority of differentially expressed proteins were shared between participants with and without STIs, with only 4 of 30 significant proteins uniquely altered in the former (Fig. 3g). Consistently, key immune markers exhibited similar expression patterns across HRY subgroups, indicating that the observed dysregulation is largely independent of concurrent STI status (Figs. 3h, i and 4). Together, these data suggest that HRY harbors a distinct immune state, defined by predicted suppression of interferon signaling and heightened inflammatory activity, consistent with a systemic response to sustained microbial or environmental triggers.

### ART reduces but does not fully resolve systemic inflammation in newly diagnosed youth

**Fig. 5:**
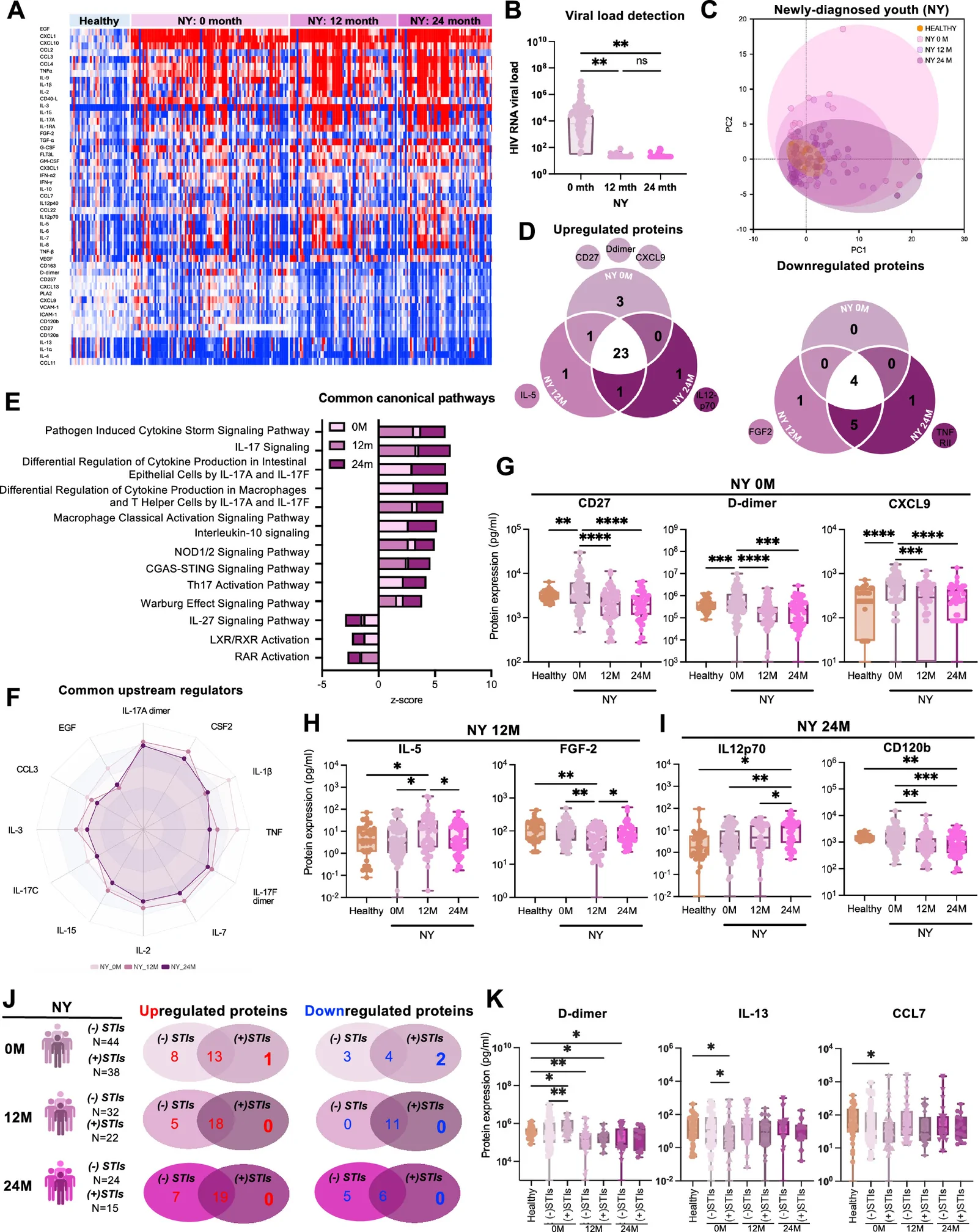
Longitudinal plasma profiling reveals overt inflammatory response in youth with HIV infection up to 24 months post diagnosis. **A** Clustered heatmap of 49 plasma immune mediators in healthy controls and newly diagnosed youth (NY) at multiple time points, annotated by antiretroviral therapy (ART) initiation timing: pre-ART (0 M), 12 months post-ART (12 M), and 24 months post-ART (24 M). **B** Plasma viral burden across time points in the NY cohort at 0 M (*n* = 92), 12 M (*n* = 59, *P* = 0.0042), and 24 M (*n* = 48, *P* = 0.0042). **C** PCA plot of plasma proteomes showing distinct profiles in healthy (yellow) and NY groups; each point represents independent samples. **D** Venn diagram of significantly upregulated and downregulated inflammatory markers shared in each cohort, with adjacent bubble plots indicating unique genes shared per group. **E** Bar plots of enriched canonical pathways upregulated or downregulated among NY groups based on gene ontology. **F** Radar plot of upstream regulators identified across NY groups; distance from center reflects normalized abundance of transcript factors. **G–I** Plasma protein profiles (pg/ml) of significantly altered immune mediators from (**D**) across healthy (brown, *n* = 89) and NY cohorts at 0 M (light pink, *n* = 62), 12 M (pink, *n* = 52), and 24 M (fuchsia, *n* = 35) tracking pre-ART (**G**), 12-months post-ART (**H**), and 24 months post-ART (**I**)*.* Individual comparisons include: **G** CD27 (0 M vs 12 M and 0 M vs 24 M, *P* < 0.0001; 0 M vs Healthy, *P* = 0.0019); D-dimer (0 M vs 12 M, *P* < 0.0001; 0 M vs 24 M, *P* = 0.0005; 0 M vs Healthy, *P* = 0.0009; CXCL9 (0 M vs 12 M, *P* = 0.0003, 0 M vs 24 M and 0 M vs Healthy, *P* < 0.0001); **H** IL-5 (0 M vs 12 M, *P* = 0.0475; 12 M vs 24 M, *P* = 0.0378; 12 M vs Healthy = 0.0251; FGF-2 (0 M vs 12 M, *P* = 0.0013; 12 M vs 24 M, *P* = 0.0442; 12 M vs Healthy, *P* = 0.0059), and **I** IL12p70 (0 M vs 24 M, *P* = 0.0022; 12 M vs 24 M, *P* = 0.0448; 24 M vs Healthy, *P* = 0.0102; CCL7 (0 M vs 12 M, *P* = 0.0051; 0 M vs 24 M, *P* = 0.0006; Healthy vs 24 M, *P* = 0.0033). Statistical significance determined using moderated *t*-tests with Benjamini–Hochberg correction for multiple comparisons. **J** Schematic representation of the stratified analysis comparing the proteomic profiles of NY cohort without (−STIs) and with (+STIs) sexually transmitted infections at baseline and subsequent timepoints relative to healthy controls. **K** Protein expression levels of upregulated (D-dimer: 0 M −STIs vs 0 M +STIs = 0.0019, Healthy vs 0 M +STIs = 0.0480, Healthy vs 12 M −STIs = 0.0014, Healthy vs 12 M +STIs = 0.0164, Healthy vs 24 M −STIs = 0.0158) and downregulated proteins (IL13: 0 M −STIs vs 0 M +STIs = 0.0442, Healthy vs 0 M +STIs = 0.0145; CCL7: Healthy vs 0 M +STIs = 0.0350) in NY +STIs, relative to healthy controls (*n* = 35) and NY −STIs at all timepoi*n*ts (0 M: −STI (*n* = 43) and +STI (*n* = 37); 12 M: −STI (*n* = 32) and +STI (*n* = 22); and 24 M: −STI (*n* = 24) and +STI (*n* = 15)). Data in (**B**), (**G**–**I**), and (**K**) are represented as boxplots with individual data points; center lines indicate medians; box boundaries indicate 25th and 75th interquartile ranges (IQR); whiskers show total range. Statistical significance for (**B**) and (**K**) determined using Brown–Forsythe and Welch ANOVA tests with post-hoc two-sided unpaired *t*-tests with Welch’s correction (*P < 0.05,**P < 0.01,***P < 0.001,****P < 0.0001). CD27 Cluster of differentiation 27, CGAS-STING cyclic GMP-AMP synthase-stimulator of interferon genes, GM-CSF granulocyte-macrophage CSF, CXCL9 C-X-C motif chemokine ligand 9, CX3CL1 C**-**X3-C motif chemokine ligand 1, EGF epidermal growth factor, FGF2 fibroblast growth factor 2, IL-13 interleukin 13, CCL7 C–C motif chemokine ligand 7, NOD 1/2 nucleotide-binding oligomerization domains 1 and 2, CD120b cluster of differentiation 120b, LXR/RXR liver X receptor/retinoid X receptor, RAR retinoic acid receptor. Diagram in (**J**) is original and was created in BioRender. Salem, G. (2026) https://BioRender.com/4ghyq72.

**Fig. 6:**
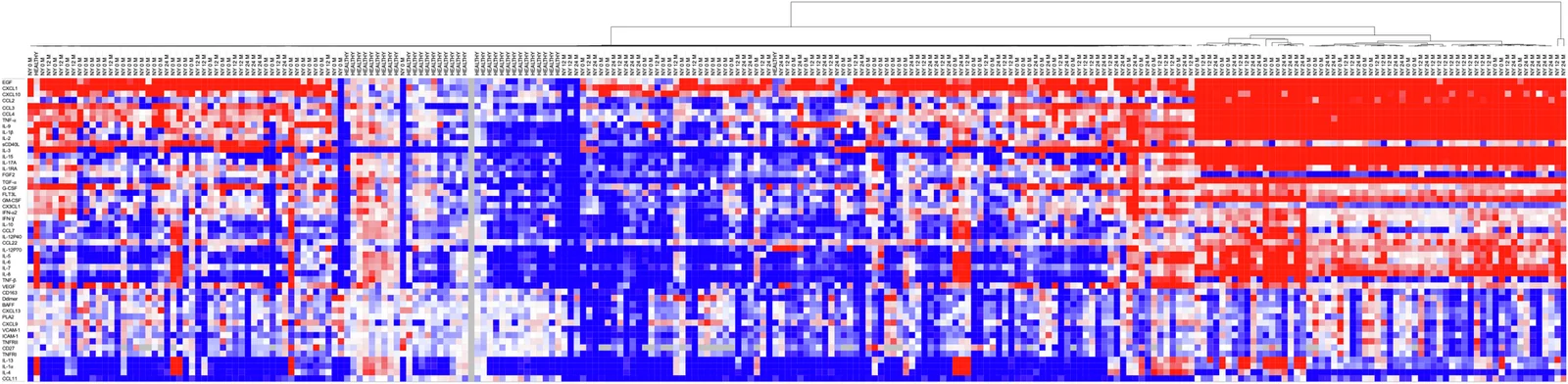
Hierarchical heatmap clustering of NY patients’ immune profiles across timepoints. Hierarchical clustering of plasma immune mediators identified to be significantly altered in newly diagnosed youth (NY) relative to healthy controls. Columns are annotated based on timing of antiretroviral therapy (ART) initiation: pre-ART (0 M), 12 months post-ART (12 M), and 24 months post-ART (24 M). Data are presented as *z*-scores in a heatmap for each plasma immune mediator.

To characterize the immunological impact of ART over time, we performed longitudinal plasma profiling in NY at baseline (0 months, immediately prior to ART initiation), and at 12- and 24-months following ART initiation. Longitudinal profiling of NY revealed dynamic changes in immune signaling following ART initiation. A heatmap of 49 immune mediators showed the highest degree of immune dysregulation in NY individuals across all time points—0 M, 12 M, and 24 M after ART (Figs. 5a and 6). Plasma viral load decreased significantly across the study period (Fig. 5b), confirming effective viral suppression. Similarly, PCA analysis also showed that NY immune profiles remained diversified across time points with overlapping to healthy controls (Fig. 5c). To further explore the persistence of inflammatory signaling, we performed a comparative analysis of differentially expressed proteins across timepoints. Venn diagram analysis revealed both shared and unique sets of dysregulated mediators at each stage of treatment (Fig. 5d). Of the 27 proteins shared among the three groups, 23 were upregulated while four were downregulated, including acute-phase proteins and interferon-stimulated genes (ISGs). Longitudinal canonical pathway enrichment analysis revealed persistent, predicted activation of innate sensing and inflammatory cascades, including cyclic GMP-AMP synthase-stimulator of interferon genes (cGAS-STING), nucleotide-binding oligomerization domains 1 and 2, and IL-17 signaling, through 24 months (Fig. 5e). Homeostatic regulators like retinoid acid receptor and liver X receptor/retinoid X receptor were consistently predicted to be inhibited, suggesting that though ART achieves virological control, it does not fully resolve the key signaling signatures driving systemic immune activation in youth. Analysis of predicted upstream regulators suggested sustained engagement of inflammatory signaling across all timepoints, including cytokine- and growth factor-associated mediators such as IL-17 family members (IL-17A dimer, IL-17F, and IL-17C), tumor necrosis factor (TNF), IL-1β, granulocyte-macrophage CSF (GM-CSF), and CCL3, as well as lymphocyte-associated cytokines (IL-2, IL-7, and IL-15) and epidermal growth factor (EGF) (Fig. 5f).

**Fig. 7:**
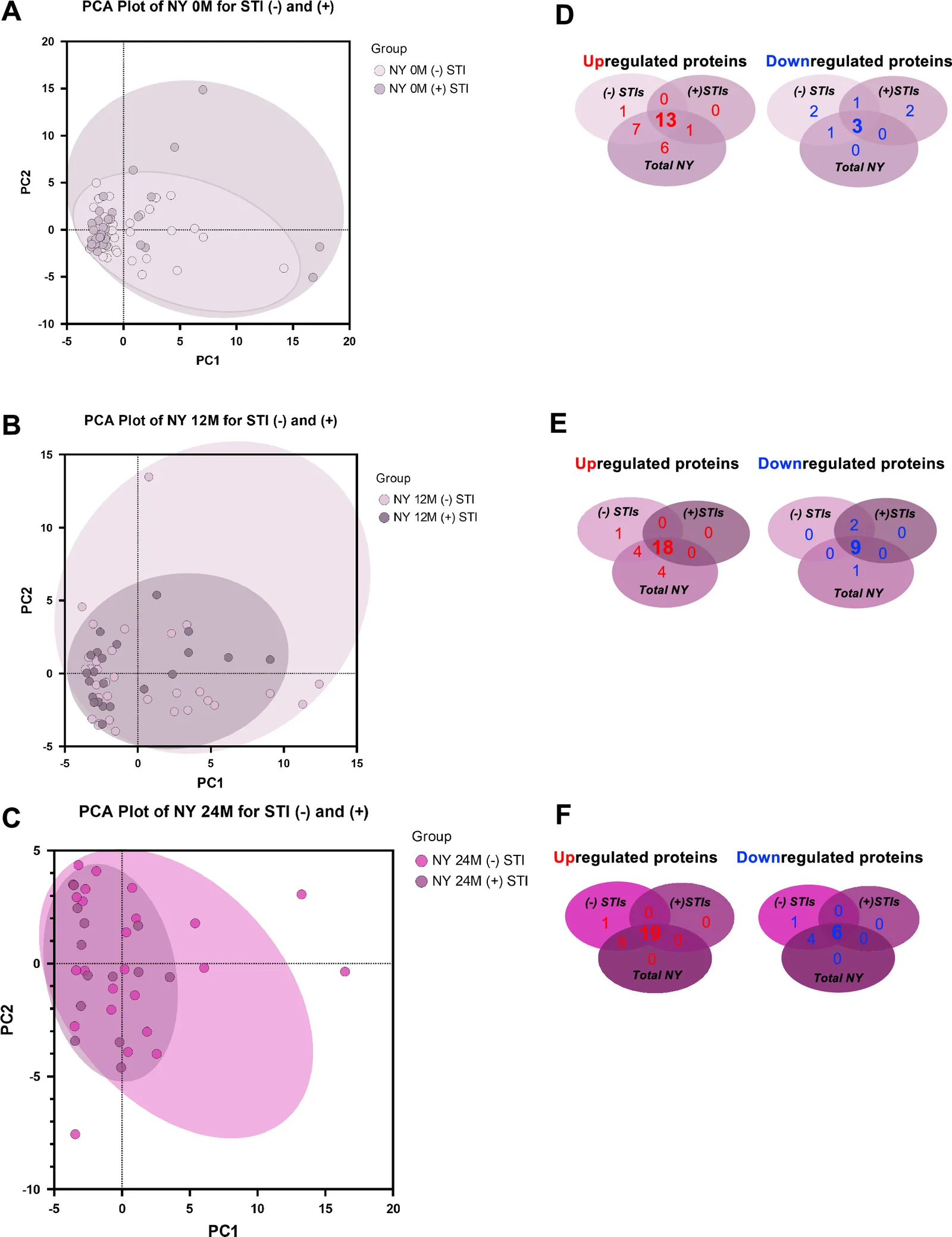
Stratified analysis of NY patients’ immune profiles based on STI status across timepoints. **A–C** PCA plot of plasma proteomes showing overlapping immune profiles of NY patients who are STI-negative (light purple) and STI-positive (dark purple) at (**A**) 0 M (−STI, *n* = 44; +STI, *n* = 38), (**B**) 12 M (−STI, *n* = 32; +STI, *n* = 22), and (**C**) 24 M (−STI, *n* = 24; +STI, *n* = 15); each point represents one sample. **D–F** Venn diagram of significantly upregulated and downregulated inflammatory markers shared between NY individuals who are STI-negative only, STI-positive only, and combined STI-negative/positive groups, relative to healthy controls at 0 M (**D**), 12 M (**E**), and 24 M (**F**).

To resolve longitudinal protein-level dynamics, we next examined significantly altered plasma immune mediators in NY and healthy controls across the three time points based on ART initiation (Fig. 5g–i). NY immune profiles remained distinct from those of healthy controls at all time points, with incomplete normalization even at 24 months after ART initiation. Given the high prevalence of STIs in the NY cohort, we stratified NY participants into those with and without concurrent STIs and performed proteomic analyses at baseline and across follow-up time points. Analyses did not demonstrate substantial differences in proteomic profiles between NY participants with and without STIs at baseline, 12, and 24 months relative to healthy controls (Fig. 7). At baseline (NY: 0 M), 3 proteins out of 31 significantly altered mediators differed by STI status (Fig. 5j, k).

### Long-term ART is associated with a distinct immune profile, but sustained predicted soluble PD-L1-mediated regulatory signatures remain

**Fig. 8:**
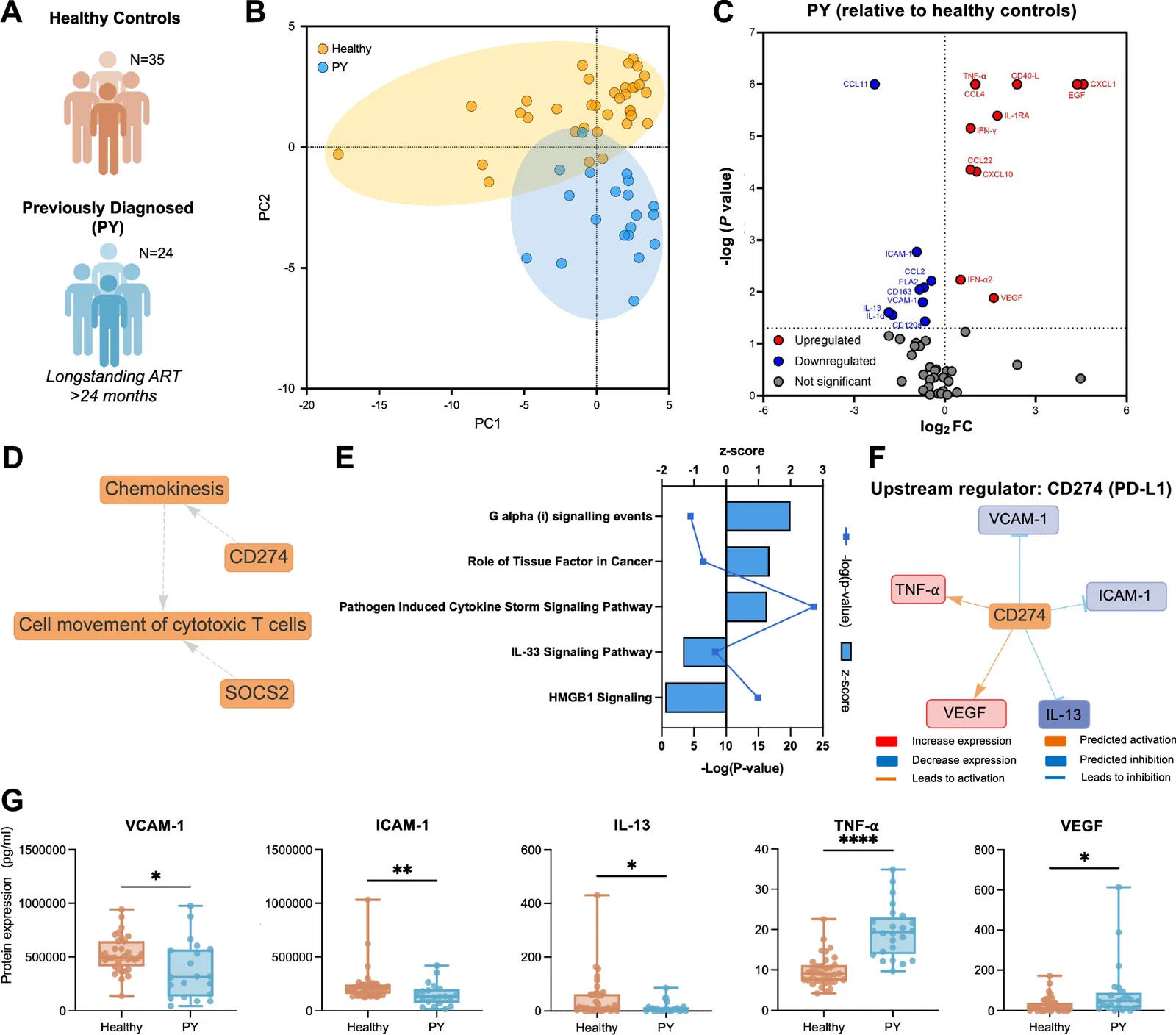
Youth living with HIV on longstanding ART exhibit resolved systemic inflammation but reveal soluble PD-L1-associated regulatory signatures. **A** Cohorts of healthy controls (*n* = 35) and previously diagnosed youth (PY, *n* = 24) on ART for at least 24 months. **B** PCA plot of plasma proteomes showing distinct immune profiles of healthy (yellow) and PY (blue) individuals; each point represents one sample. **C** Volcano plot of global plasma proteomics highlighting proteins significantly upregulated (red) or downregulated (blue) in PY relative to healthy controls (*P* < 0.05 and log_2_ fold-change (FC) > 1.1 or <1.1); the horizontal dotted line indicates *P* value cut-off. Statistical significance was determined using two-tailed Mann–Whitney tests for multiple comparisons without correction. **D** Network diagram of biological pathways and upstream regulators enriched in the PY group. **E** Bar plots of enriched canonical pathways commonly up or downregulated in PY group, based on gene ontology. **F** Graphical summary of transcription factors regulating CD274 (PD-L1) expression; **G** Protein expression (pg/ml) profiles of significantly altered plasma immune mediators (VCAM-1, *P* = 0.0158; ICAM-1, *P* = 0.0017; IL-13, *P* = 0.0247; TNF-α, *P* < 0.0001; and VEGF-A, *P* = 0.0131) among healthy controls (brown, *n* = 35) and PY group (blue, *n* = 24). Data points represent individual participants; boxes indicate the 25th and 75th interquartile ranges (IQR) with the median line, and whiskers extend to the minimum and maximum values. Statistical significance was determined using two-tailed Mann–Whitney test; *P < 0.05, **P < 0.01, ****P < 0.0001. CD274 Cluster of differentiation 274, HMGB1 high mobility group box 1, ICAM-1 intercellular adhesion molecule 1, IL-33 interleukin 33, PD-L1 programmed death-ligand 1, SOCS2 suppressor of cytokine signaling 2, TNF-α tumor necrosis factor alpha, VCAM-1 vascular cell adhesion molecule 1, VEGF-A vascular endothelial growth factor A. The illustration generated in (**A**) is original and was created in BioRender. Salem, G. (2026) https://BioRender.com/4ghyq72.

We next assessed systemic immune profiles in PY to determine if long-standing ART restores systemic homeostasis relative to healthy controls (Fig. 8a). PCA showed a clear separation between healthy controls and the PY group with minimal overlaps of selected plasma immune signatures (Fig. 8b). We next identified specific markers driving this divergence and found significant dysregulation of several key pathways in the PY group relative to healthy controls. PY individuals exhibited significantly higher levels (adjusted *P* < 0.05, |log₂FC| > 1.1) of several pro-inflammatory cytokines and chemokines (TNF-α, IFN-γ, CD40 ligand (CD40-L), CXCL9, and CXCL10), while certain factors were significantly lower in the PY group (CCL11, intercellular adhesion molecule 1 (ICAM-1), vascular cell adhesion molecule 1 (VCAM-1), CCL2, and CD163), indicating residual immunomodulatory activity despite viral suppression (Fig. 8c). Functional enrichment analysis revealed that the differentially expressed proteins were associated with altered leukocyte dynamics, with an observed predicted activation of chemokinesis and the cell movement of cytotoxic T-cells, involving key nodes such as soluble CD274 (or programmed death ligand 1, PD-L1)^31^ and suppressor of cytokine signaling 2 (Fig. 8d). This was further supported by canonical pathway analysis (Fig. 8e), which identified G alpha (i) signaling events and the role of tissue factor in cancer as highly activated (*z*-score >1). Conversely, the high mobility group box 1 and IL-11 signaling pathways were significantly predicted to be inhibited (*z*-score <−1), despite their high statistical significance (−log(*P* value)), suggesting a targeted suppression of specific alarmin-mediated inflammatory responses in the PY group. CD274 was identified as a significant upstream regulator, with predicted upregulation of pro-inflammatory factors, such as TNF and vascular endothelial growth factor A (VEGF-A) (Fig. 8f). CD274 was also linked to a predicted inhibition of IL-13 and decreased expression of adhesion molecules (VCAM-1 and ICAM-1).

Quantitative protein expression analysis demonstrated that pro-inflammatory mediators, such as TNF (*P* < 0.0001) and angiogenic factor VEGF-A, remained elevated in the PY group relative to healthy controls, despite overall shifts toward baseline for other markers (Fig. 8g). In contrast, PY individuals exhibited significantly lower levels of adhesion molecules, VCAM-1 (*P* < 0.05) and ICAM-1 (*P* < 0.01), alongside reduced IL-13 compared to healthy controls. These findings suggest that long-term ART, while effective in modulating many aspects of immune function, may be accompanied by persistent TNF-driven inflammation and altered vascular signaling signatures.

### Comparative analysis identifies shared and stage-specific immune mediators across the HIV continuum

**Fig. 9:**
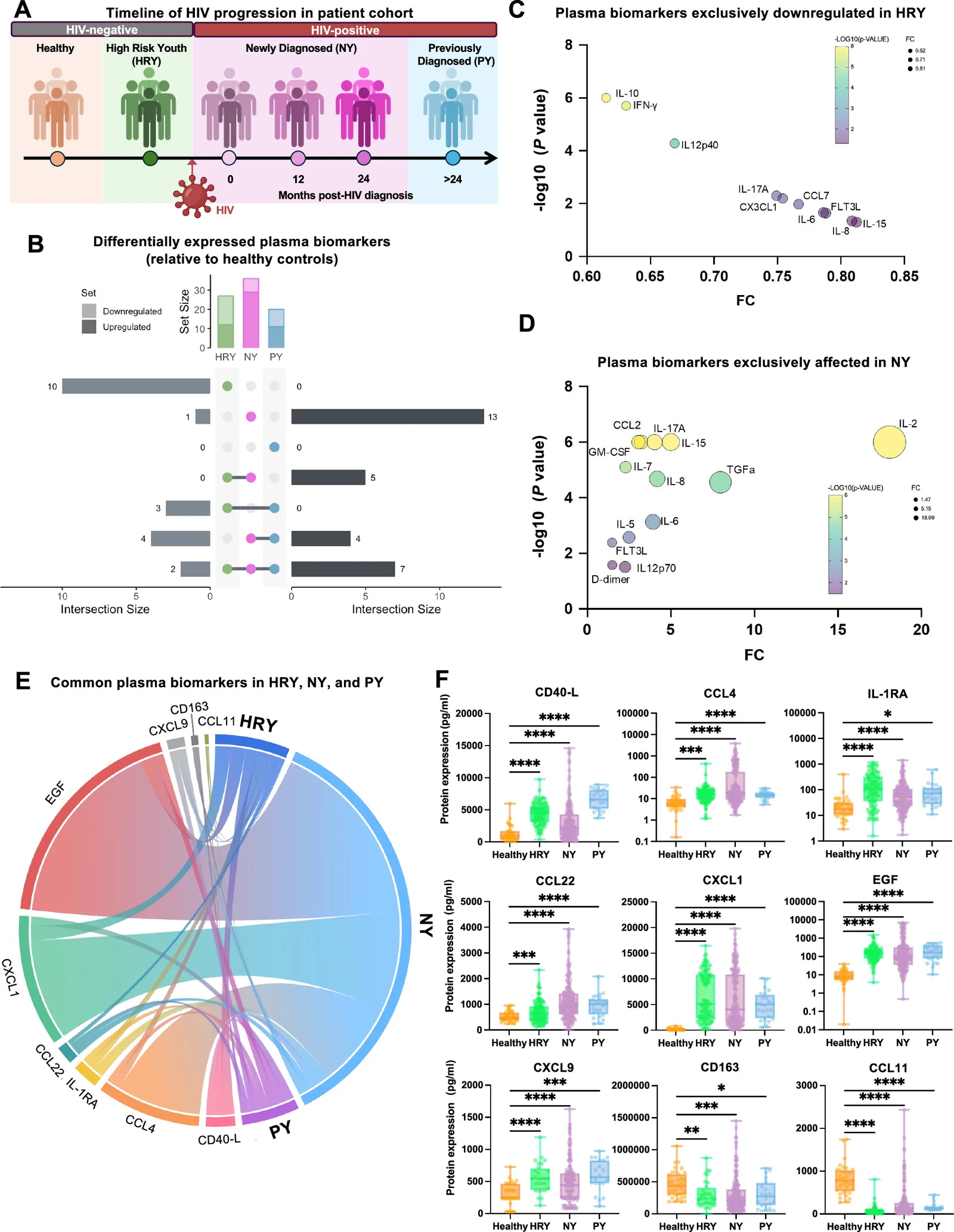
Comparative analysis identifies key plasma biomarkers associated with risk of HIV infection and HIV-associated disease progression. **A** Timeline of HIV progression in the study cohort, including groups without HIV infection (healthy controls and high-risk youth, HRY) and groups with established with HIV diagnosis (newly diagnosed, NY; previously diagnosed, PY). NY participants were followed longitudinally at three time points relative to ART initiation: baseline or prior to ART (0 M), 12 months (12 M), and 24 months (24 M). Previously diagnosed youth (PY) group is on ART for at least 24 months. **B** UpSet plot of differentially expressed plasma biomarkers in HRY (green), NY (pink), and PY (blue) groups compared with healthy controls, illustrating unique and shared upregulated (dark gray) or downregulated (light gray) proteins. **C**, **D** Enrichment plots of plasma biomarkers exclusively downregulated in HRY (**C**) or exclusively affected in NY (**D**). Statistical significance for (**C**, **D**) was determined using two-tailed Mann–Whitney test for multiple comparisons without correction. Circle size reflects fold-change magnitude relative to healthy controls, while the color scale indicates the significance of enrichment. **E** Circular plot illustrating differentially expressed plasma biomarkers and their shared relationships among HRY, NY, and PY groups. **F** Plasma protein profiles (pg/ml) of commonly shared immune mediators in (**E**) from across HRY (*n* = 110), NY (*n* = 208), and PY (*n* = 24) groups relative to healthy (*n* = 35) controls. Individual markers were assessed: CD40-L (Healthy vs HRY, NY or PY, *P* < 0.0001; CCL4 (Healthy vs HRY, *P* = 0.0007; Heathy vs NY or PY, *P* < 0.0001); IL-1RA (Healthy vs HRY or NY, *P* < 0.0001; Healthy vs *P*Y, *P* = 0.0172); CCL22 (Healthy vs HRY, *P* = 0.0006; Healthy vs NY or PY, *P* < 0.0001); CXCL1 (Healthy vs HRY, NY or PY, *P* < 0.0001); EGF of Healthy vs HRY, NY or PY, *P* < 0.0001); CXCL9 (Healthy vs HRY or NY, *P* < 0.0001; Healthy vs PY, *P* = 0.0002); CD163 (Healthy vs HRY, *P* = 0.0029; Healthy vs NY, *P* = 0.0002; Healthy vs PY = 0.0152); CCL11 (Healthy vs HRY, NY or PY, *P* < 0.0001). Dots represent individual participants; box plots show the median line and the 25th and 75th interquartile ranges (IQR); whiskers extend to minimum and maximum values. Statistical significance determined using Brown–Forsythe and Welch ANOVA tests followed by a post-hoc two-sided Dunnet’s T3 multiple comparison’s test with individual variances computed for each comparison (*P < 0.05, **P < 0.01, ***P < 0.001, ****P < 0.0001). CD163 Cluster of differentiation 163, CCL4 C–C motif chemokine ligand 4, CXCL9 C-X-C motif chemokine ligand 9, EGF epidermal growth factor, FC fold change, FLT3L Fms-related tyrosine kinase 3 ligand, GM-CSF granulocyte-macrophage colony-stimulating factor, IL-1RA interleukin 1 receptor agonist, CD40-L soluble CD40 ligand, TGF-α tumor growth factor alpha. The illustration generated in (**A**) is original and was created in BioRender. Salem, G. (2026) https://BioRender.com/4ghyq72.

To identify circulating biomarkers associated with HIV risk, progression, and long-term treatment, we compared the plasma proteomes of HRY, NY, and PY groups relative to healthy controls (Fig. 9a). Differential expression analysis revealed that the NY cohort exhibited the largest set of uniquely upregulated proteins (*n* = 13), reflecting acute immune activation at the time of HIV diagnosis (Fig. 9b). While a core set of 7 upregulated and 2 downregulated proteins was shared across all groups, the HRY group was primarily characterized by an exclusive suppressive profile (*n* = 10 uniquely downregulated proteins). In HRY individuals, we observed selective downregulation of key homeostatic and pro-inflammatory mediators, with IL-2 and IL-8 exhibiting the most significant decrease (−log10 *P* > 5.5) (Fig. 9c). Other markers with exclusive downregulation in this group included CCL2, IL-7, and tumor growth factor alpha (TGF-α), suggesting a baseline state of immune suppression in the high-risk population. Conversely, the NY group was defined by a robust inflammatory signature consistent with acute infection, with the striking upregulation of IL-2 (fold change ≈ 18), accompanied by significant elevations in IL-17A, CCL2, and IL-15 (all −log10 *P* ≈ 6) (Fig. 9d). Notably, markers of systemic coagulation (D-dimer) and myeloid signaling (Fms-related tyrosine kinase 3 ligand, FLT3L) were also exclusively elevated in the NY cohort. Together, these findings suggest that while the HRY group is characterized by a potential deficit in critical T-cell and chemotactic signals, the NY cohort is dominated by a rapid, large-scale expansion of Th1- and Th17-related cytokines.

Chord diagram analysis further visualized the shared biomarkers across groups, identifying a central cluster of mediators, including EGF, CXCL1, CCL4, and CD40-L, that remain significantly altered regardless of infection stage or treatment status (Fig. 9e). EGF and CXCL1 showed the highest connectivity between the NY and PY groups, while other mediators, including CD163, CXCL9, and CCL11, primarily linked the HRY and NY cohorts. Consistent with these network patterns, plasma levels of CD40-L, CCL4, IL-1RA, CCL22, CXCL1, EGF, and CXCL9 remained significantly elevated across all groups compared with healthy controls, whereas CD163 and CCL11 were consistently reduced (Fig. 9f). Collectively, these results define a conserved circulating immune signature that spans the trajectory from HIV susceptibility (HRY), to acute infection (NY), and long-term viral suppression (PY).

## Discussion

In this study, we leveraged high-dimensional plasma proteomics and viral load quantifications in a well-characterized cohort of adolescents and young adults, including healthy controls, high-risk HIV-negative individuals, newly diagnosed patients monitored longitudinally through 24 months of ART, and youth on long-term therapy. It is important to note that despite significant advancements in ART, HIV remains a pressing health issue among adolescents and young adults due to their unique immunological and behavioral vulnerabilities^2,4^. Our longitudinal proteomic analyses across high-risk uninfected youth (HRY), newly diagnosed (NY), and previously diagnosed youth on long-term ART (PY) provide novel insights into the immune perturbations that characterize HIV susceptibility, disease progression, and partial restoration of circulating protein homeostasis. These shared markers point to common circulating immunologic pathways that may underlie both HIV susceptibility and disease progression in youth. Overall, these findings highlight a combination of unique and overlapping immune signatures across the HIV infection spectrum, suggesting that a core inflammatory program is engaged early in high-risk individuals and sustained, albeit variably, across stages of infection and treatment. These findings have important implications for biomarker development, risk stratification, and novel therapeutic strategies tailored to this vulnerable population.

We identified a distinct immune dysregulation pattern in HRY characterized by suppressed interferon signaling and dysregulated innate responses linked to heightened microbial activation. This altered immune landscape suggests that chronic, subclinical immune activation and impaired antiviral defenses may represent a perturbed systemic profile that precedes HIV acquisition, potentially rendering these youth more susceptible to infection^32^. These findings align with observations that microbial translocation and persistent inflammation can prepare the immune environment for viral entry and replication^33–35^, as seen in early studies of adults at-risk of HIV, where systemic activation was a precursor to seroconversion ^36–38^.

A key finding in our cohort was that while clinical STIs (e.g., syphilis, gonorrhea, or chlamydia) were present in the HRY group, the observed immune signatures were largely independent of concurrent STI status. While STIs such as syphilis are known to increase the risk of sexual and vertical transmission of HIV by two-to-five-fold through the upregulation of HIV-tropic surface receptors^39,40^, our data suggests a more fundamental baseline dysregulation in HRY. This implies that environmental triggers beyond active STIs may influence these profiles. Because the current work lacks quantitative metadata to directly evaluate psychosocial stress, invoking such lifestyle factors remains entirely speculative. However, it is well-established that unmeasured, non-infectious confounding variables, including metabolic shifts such as obesity-induced inflammation or chemical exposures like tobacco smoke, can profoundly alter systemic inflammatory networks independently of active viral or bacterial infection^41,42^. Our data cannot rule out these critical environmental and systemic confounders, which represent a key boundary of our observational cohort study.

Intriguingly, the downregulation of 10 proteins in HRY individuals, many with pro-inflammatory properties, suggests a more nuanced immune landscape than a simple “high-versus-low” activation model. While immune activation is a well-recognized feature of HIV pathogenesis and can act as a double-edged sword, the specific suppression of selected pro-inflammatory mediators in HRY, alongside the predicted activation of NF-κB and STAT3 pathways, may reflect a highly nuanced, non-linear immune landscape. While a blunted innate sensing signature could theoretically impair rapid effector cell recruitment, this profile may be simultaneously countered by the robust elevation of circulating β-chemokines such as CCL3 and CCL4. Because these specific proteins function as competitive inhibitors of the CCR5 co-receptor^29,30^, their systemic upregulation could paradoxically serve as a localized or protective mechanism designed to block HIV entry. This underscores that the immunobiological state of HRY may not only be a simple model of global impairment, but rather a complex, dual-natured systemic signature where competitive viral entry barriers could coexist alongside altered innate sensing pathways. While our proteomic data cannot establish cellular functional capacity, this altered profile correlates with pathways involved in effector cell recruitment or protective antiviral states upon HIV exposure, warranting further functional investigation to explore its relationship with infection susceptibility. However, the relationship between immune activation and HIV acquisition is complex and warrants further functional investigation. Clinically, these findings emphasize the need for early immune profiling to identify high-risk biological signatures, enabling more targeted interventions, such as enhanced pre-exposure prophylaxis (PrEP) counselling or therapies designed to restore innate antiviral defenses before exposure occurs ^43–45^.

Among newly diagnosed youth, systemic inflammation marked by activation of cytokine pathways persisted despite two years of ART and robust viral suppression. Persistent engagement of inflammatory signaling signatures, such as IL-17 family members and lymphocyte-associated cytokines, suggests incomplete normalization of the plasma proteome following ART and may contribute to elevated checkpoint-associated circulating signatures and increased risk of HIV-associated non-AIDS comorbidities, including cardiovascular disease and neurocognitive impairment^10,11,46–48^. We have expanded our analysis to address this cohort heterogeneity, explicitly noting that the increased dispersion observed in the longitudinal cohort at 0 M likely reflects baseline biological heterogeneity prior to the stabilization of proteomic footprint under long-term ART. This noted slow resolution of immune activation following ART initiation in newly diagnosed HIV infection underscores the potential importance of long-term ART and concurrent treatment of STIs in the clinical care of YLWH infection. These findings parallel adult cohorts but underscore the importance of early therapeutic interventions in youth, whose immune systems may be plastic yet vulnerable to long-term complications^49,50^. While functional validation is required before clinical translation can be considered, these predictive proteomic trends highlight areas where future research might explore the utility of adjunctive anti-inflammatory or immune-modulatory concepts alongside ART, such as statins, cytokine blockers, or agents targeting microbial translocation, to fully restore immune homeostasis and reduce chronic inflammation^35,49,51^. In fact, studies in adults with HIV show elevated inflammatory markers such as IL-4, IL-6, and IL-10, along with elevations of TNF-α and TGF-β to be significantly elevated in patients clinically diagnosed with HIV before ART therapy. With 12 months of therapy, IL-4, IL-6, and IL-10 returned to normal levels, whereas TNF-α and TGF-β were persistently elevated^7^. It appears that this reduction correlated with ARTs are seen mostly within the first 6–12 months after starting therapy^6^. Other studies have described accelerated aging in children with perinatally acquired HIV, with implications for renal, cardiac, endocrine, bone mineral, and neuropsychological conditions as well as increased cancer risk^12^.

In previously diagnosed youth on long-term ART, immune profiles largely normalized relative to healthy controls but with persistent expression of immune checkpoint markers like soluble PD-L1 (sPD-L1), which represents a predictive checkpoint-associated signature^52^. While cell-surface PD-L1 acts as a compensatory inhibitory signal, the precise systemic role of its soluble form remains to be fully elucidated. However, elevated sPD-L1 frequently tracks with chronic inflammatory states, diminished vaccine responses, and co-infection responsiveness^52,53^. Though carefully timed checkpoint blockade or immune rejuvenation strategies can be explored^54^, these circulating features warrant further exploration regarding whether cell-surface checkpoint pathways are altered, though any potential therapeutic strategies remain purely speculative at this stage. However, the risks of immune activation must be balanced against potential benefits, highlighting the need for controlled clinical trials in this population^32^.

Finally, our comparative plasma proteome analyses revealed a transition from a distinct, suppressive immune profile in HRY to a hyper-inflammatory state upon acute infection (NY), driven by elevated Th1/Th17 cytokines. While early infection causes a massive cytokine storm, a core set of persistent markers, including CD40-L, CCL4, and EGF, remains altered across all stages, indicating long-term immune dysregulation. This “stable dysregulation” suggests that while ART manages acute-phase spikes, underlying immune network connectivity trends appear chronically impaired. Thus, early identification of individuals with persistent inflammation or immune dysregulation could guide personalized interventions, improve clinical outcomes and inform public health strategies^33^.

In summary, our findings highlight that HIV risk, infection, and progression in youth are marked by complex immune perturbations that are incompletely reversed by cART. This underscores the urgent need for integrated clinical strategies combining ART with targeted immunotherapies and precision monitoring to achieve a durable immune restoration and improve long-term health outcomes in this vulnerable population.

### Limitations and future directions

While our study provides comprehensive proteomic insights, there are limitations which warrant consideration. A key study limitation is its reliance on circulating protein panels to infer upstream regulator and the absence of mucosal or tissue-resident immune profiling. Furthermore, plasma protein concentrations reflect a complex net outcome of systemic clearance kinetics, tissue compartmentalization, receptor shedding, and target-cell consumption, rather than a direct measurement of intracellular molecular pathways. While coordinated directional shifts in the 49 plasma protein concentrations could strongly predict specific upstream transcriptional regulators, such as cGAS-STING and STAT3 via IPA modeling, we explicitly acknowledge that IPA upstream regulator analyses do not provide direct evidence of localized intracellular activation or inhibition, particularly when the analytical input is restricted to a panel of circulating proteins. Hence, these findings must be interpreted strictly as hypothesis-generating signatures rather than confirmed intracellular signaling states. We also emphasize that though our current study probed soluble checkpoint molecules, such as sPD-L1, no functional cellular assays were performed and that these circulating proteomic features are not sufficient proxies to define true cellular T cell exhaustion or dysfunction. To definitively map these intracellular signaling states and validate whether these signatures reflect authentic immune states, future studies should integrate orthogonal intracellular validation in localized targets and from fresh PBMCs. Additionally, behavioral and socio-environmental variables, which may influence immune signatures, were not deeply characterized in the current work. Future studies must investigate within the defined memory and activation context, and should include high-resolution single-cell and spatial immune profiling (including PD-1)^55^, dye-dilution T-cell proliferation assays (via cell-tracking dyes)^56^, cytokine production profiling after ex vivo stimulation (using anti-CD3/CD28 or ionomycin stimulants)^57^, intracellular signaling readouts using mass cytometry (e.g., cytometry by time-of-flight (CyTOF) mapping of pSTAT3)^58^, metabolomics, and longitudinal behavioral data to develop a holistic understanding of HIV-related immune trajectories in youth. Collectively, our findings underscore that immune responses to HIV exposure and infection in youth are not simply scaled-down versions of adult responses but instead represent a unique immunobiological program shaped by development, chronic risk exposure, and treatment response. The observational nature of the current study precludes causal inferences; hence, interventional trials evaluating immune-modulatory agents alongside ART in youth would be needed to translate these findings to clinical practice. Understanding these dynamics will be essential for optimizing interventions, particularly in the long-term care of adolescents living with and at risk for HIV.

## Methods

### Ethical approval

The study was approved by the UCLA Institutional Review Board, which was the overseeing IRB and the IRBs of participating sites, with written informed consent obtained from all participants. For the main study, participants were paid $50 for each visit. This is a secondary analysis on data obtained from those visits and no additional compensation was given.

#### Study cohorts and sample collection

Youth aged 12–24 were enrolled in the CARES study, sponsored by the NIH ATN, U19 grant (U19HD089886), which recruited youth at the highest risk of acquiring HIV, including gay, bisexual, transgender, and homeless youth, and YLWH in the Los Angeles and New Orleans areas^59^. Youth enrolled in ATN studies in the U.S. are representative of young individuals living with or at risk for HIV acquisition across the country^25^. Participants were recruited and screened from gay-identified community-based organizations, homeless shelters, adolescent medical clinics, as well as social media, dating apps, and referrals. Participating ATN147 study sites include David Geffen School of Medicine at the University of California at Los Angeles, Department of Pediatrics, Division of Infectious Diseases, the Los Angeles lesbian, gay, bisexual, and transgender (LGBT) Center, and Tulane University School of Medicine, Department of Adolescent Medicine. Along with screening for participants without HIV infection, participants who tested positive for HIV were recruited through referral from the ATN149 CARES high-risk cohort without HIV and direct referrals from the community. From these screenings, a cohort including 103 recently diagnosed HIV treatment naïve youth (ATN 147)^23,60^, 170 youth with established HIV infection (ATN 148)^24,61^, and 1487 youth without HIV infection (ATN 149) was recruited and enrolled^22,62^. Recruitment included screenings from community-based outreach programs and participating agencies. ATN 147 and 148 were YLWH^23,24,59–61^, and ATN 149 were youth without HIV infection but with multiple risk factors for acquiring HIV^22,62^. Participants were enrolled between 2017 and 2020 and followed for 2 years in each one of the corresponding ATN CARES studies. The present proteomic analysis evaluated plasma specimens from a subset of participants enrolled in these studies.

#### Study inclusion and exclusion criteria

Overall inclusion criteria for enrollment in ATN CARES studies included: (1) age of 12 to 24 years, and (2) ability and willingness to provide written informed consent. Exclusion criteria included (1) active drug or alcohol use or dependence that, in the opinion of the site investigator, would interfere with adherence to study requirements, (2) any acute, chronic, or recent and clinically significant medical condition that, in the opinion of the site investigator, would interfere with adherence to study requirements or jeopardize the safety or rights of the participant, (3) chronic or recurrent use of medications that modify host immune response, for example, oral or parenteral steroids and cancer chemotherapy, and (4), being over the age of 24 or younger than 12 years. Additional individual inclusion or exclusion criteria specific to the focus of each ATN study (147, 148, or 149) were present^22–24^. Participants enrolled in the HIV cohorts had confirmatory HIV testing performed by review of medical records and rapid screening for HIV-1/2 antibodies and HIV-1 p24 antigen in blood using Alere 4th generation Determine strips on enrollment. HIV ribonucleic acid (RNA) levels were measured at every study visit. Participants enrolled in ATN 149 (high-risk cohort) had rapid screening for HIV-1/2 antibodies and HIV-1 p24 antigen in blood with Alere 4th generation Determine strips at each study visit, and performance of confirmatory HIV-1 RNA quantitative polymerase chain reaction (PCR) on Cepheid’s GeneXpert point of care machines following each study visit.

#### Other covariates

Data collection for all participants was recorded at baseline, as previously described in ref. ^59^. A detailed questionnaire regarding demographic factors such as age, race/ethnicity, gender, and sexual orientation, plus behavioral elements including sexual history and practices, drug and alcohol use, was administered. Drug use was classified as ever versus never use in the past four months.

Control participants were recruited from participants in a study of first-line responders during the COVID-19 pandemic conducted at UCLA. They were all male individuals, in good health, without HIV infection or concurrent STIs, who worked as frontline workers (e.g., firefighters, physicians, nurses) in Los Angeles, CA between 2020 and 2022, aged 20–27 years of age ^63^.

For the inflammatory marker profile study, we evaluated specimens from 93 of 103 ATN 147 participants obtained at baseline, specimen availability being the only criterion for selection of participants at baseline in ATN 147. None of the participants had an undetectable virus load at that time point, as they had not yet initiated ARTs, and they had just been diagnosed with HIV. The presence of an undetectable viremia was the selection criterion used for specimens obtained at 12 and 24 months following participant cART initiation. At the 12- and 24-month timepoints, we evaluated specimens from 63 and 56 ATN 147 participants, respectively, with IPA performed only on specimens of patients who achieved an undetectable viral load (undetectable was defined as less than 20 copies/ml of plasma, which was the lower limit of detection) by that timepoint. The 24 participants originating from ATN 148 who were evaluated in the present study all had sustained undetectable viral loads during the study and at the time point their specimen was obtained; having a suppressed virus load plus specimen availability were the criteria used for specimen selection in this group. With IPA performed only on specimens of participants who achieved an undetectable plasma viral load following ART initiation in ATN 147 and 148, we aimed to minimize confounding alterations in systemic protein profiles directly driven by active HIV viremia. For ATN 149, specimens were selected based on sample availability in our specimen repository.

#### Plasma proteomics profiling

Plasma samples were collected at study visits, processed, and stored at −70 °C during the conduct of their respective studies. For this study, samples were thawed and subjected to a high-throughput multiplex bead-based immunoassay (Luminex platform) panel targeting 49 immune-related proteins, including cytokines, chemokines, and soluble receptors (Supplementary Table 2). Analytes, including IL-6, CD163, D-dimer, CD257 (or B-cell activating factor), CXCL13, phospholipase A2, CXCL9, VCAM-1, ICAM-1, CD120a, and CD120b (or TNF receptors I and II, respectively, TNFRI, and TNFRII), and CD27, were measured in duplicate by R&D’s bead-based immunoassays on a MAGPIX system. Additional analytes, such as EGF, fibroblast growth factor 2 (FGF2), CCL11 (or eotaxin), TGF-α, G-CSF, FLT3L, GM-CSF, C-X3-C motif chemokine ligand 1 (CX3CL1 or fractalkine), IFN-α2, IFN-γ, CXCL1 (or growth-regulated oncogene), IL-10, CCL7 (or monocyte chemotactic protein 3), IL12p40, CCL22 (or macrophage-derived chemokine), IL12p70, IL-13, IL-15, CD40-L, IL-17A, IL-1RA, IL-1α, IL-9, IL-1β, IL-2, IL-3, IL-4, IL-5, IL-7, IL-8, CXCL10 (or interferon gamma-induced protein 10 kDa, IP-10), CCL2 (or monocyte chemoattractant protein 1), CCL3 (or macrophage inflammatory protein 1 alpha, MIP-1α), CCL4 (or MIP-1β), TNF-α, TNF-β, and VEGF-A, were quantified using a Millipore multiplex panel on an LX200 system. All measurements represent absolute protein concentrations, expressed in pg/ml. Data were acquired using Luminex xPONENT® software, and values were normalized to internal controls to account for batch and inter-assay variability. Specifically, to minimize technical variability, samples were randomized across assay plates, and identical quality control (QC) samples and calibration standards were included on each plate provided by the manufacturer (Millipore). Intra-assay and inter-assay coefficients of variation (CVs) were monitored, and analytes with excessive technical variability (e.g., CV > 20–30% or low bead counts) were excluded according to predefined quality control criteria. Plate effects were assessed using PCA and mixed-effects models and adjusted where necessary. Additionally, because each Luminex plate accommodates 38–40 samples, three bridging controls were included on every plate to control for plate-to-plate variation and ensure accurate normalization across the entire batch of 262 analyzed specimens.

#### Viral load quantification

HIV viral loads were measured using HIV-1 RNA quantitative real-time PCR at Quest Diagnostics in ethylenediaminetetraacetic acid plasma at the time of the conduct of the original ATN studies, with data entered in a RedCap database. Viral loads were expressed as copies per milliliter, with 20 copies/ml of HIV RNA being the lower limit of detection and 10^7^ copies/ml the upper limit of detection.

#### Data processing and statistical analysis

Principal Component (PC) PCA was performed using GraphPad Prism (v10.6.1). Prior to PC analysis, protein concentrations were *z*-score normalized across comparison groups by subtracting the group mean concentration value and dividing by the standard deviation, resulting in a mean of 0 and sample standard deviation of 1. Because all samples were processed simultaneously with internal controls, batch effects were absent, and batch correction was omitted. Global proteomic trajectories across groups and time points were visualized by plotting the first two PCs (PC1 and PC2) to account for the maximum amount of variation in the data. Differential expression analyses between cohorts (e.g., HRY vs. healthy controls, NY vs. healthy controls) was evaluated using moderated *t*-tests with *P* values adjusted using the Benjamini–Hochberg method for multiple comparisons^64^. Proteins with adjusted *P* values < 0.05 and an absolute fold-change thresholds (|FC| > 1.1) were defined as significantly differentially expressed. Volcano plots, heatmaps, Venn diagrams, and UpSet plots were generated using in *R* using ggplot2 and Complex Heatmap^65^. Conceptual workflow timelines and stratified analytical schematics were created by the authors using BioRender.com under an Academic Subscription (License Agreement numbers: Fig. 1A, RH2A3JSFKK; Fig. 3A, QH2A3KEH4G; Fig. 3G, IQ2A3KEH9P; Fig. 5J, RL2AKEHEW; Fig. 8A, DB2A3KEHIJ; and Fig. 9A, VJ2A3KEHP7).

For multiple-cohort profiles with unequal variances, global significance was determined using Brown–Forsythe and Welch ANOVA tests followed by post-hoc two-sided Dunnett’s T3 multiple comparison tests. Skewed distributions were evaluated using two-tailed Mann–Whitney tests or Kruskal–Wallis tests with two-sided post-hoc Dunn’s multiple comparisons tests. Group variance heterogeneity was assessed via Multivariate Homogeneity of Groups Dispersion (PERMDISP) in *R* using the vegan package. Statistical significance levels across all figures are indicated by asterisks.

#### Ingenuity pathway analysis (IPA)

To generate mechanistic hypotheses regarding upstream molecular pathways from protein dynamics, functional enrichment, GO annotation, canonical pathway analysis, and upstream regulator prediction were performed using Ingenuity Pathway Analysis (IPA, build version 127006219, Ingenuity Systems; Qiagen Inc.)^66^. Specifically, log-transformed absolute concentrations and their corresponding differentially expression values (log_2_ fold-change and Benjamini–Hochberg adjusted *P* values) were analyzed using the IPA Core Analysis module. Downstream and canonical pathway analyses were performed alongside Transcriptional Regulator Analysis to identify potential regulatory nodes (e.g., NF-κB, STAT3, and cGAS-STING) driving the observed extracellular profiles. Overlap *P* values were calculated using a right-tailed Fisher’s exact test (*P* < 0.05) to assess the statistical significance of protein enrichment. The analysis identified significantly enriched biological pathways and predicted upstream transcriptional regulators potentially responsible for observed expression patterns. All GO and pathway analyses were conducted within this platform using standard settings; no independent external pipeline was used.

Activation z-scores were utilized to infer the directional status of the regulators as either activation (≥ 2) or inhibition (≤−2) by comparing the concordance between observed molecular changes and curated literature-based causal relationships based on directionally coordinated downstream targets in the IPA Knowledge Base. To ensure analytical rigor and minimize potential technical bias, samples from across timepoints were randomized across processing batches, and data were subjected to robust normalization prior to IPA input. Upstream regulator analysis was similarly performed to identify candidate molecules potentially driving the observed expression patterns, though these predictions are inferred computationally and provide a statistical inference of the likelihood of pathway activation based on the directionality of the proteome rather than a direct measurement of pathway flux or proteomic data. Analyses were based on fold-change values calculated relative to healthy controls, and graphical summaries and network diagrams were generated within the IPA platform using standard settings. Because these analyses were extrapolated strictly from a 49 circulating plasma protein panel, all mapped pathways and upstream regulator states are defined and interpreted as predictive, exploratory, and hypothesis-generating rather than definitive measures of localized intracellular signaling transcription factor activity.

#### Multivariate homogeneity of dispersion

Multivariate homogeneity of groups dispersion (PERMDISP) analysis was conducted in *R*^67^ using the vegan package. Specifically, a Euclidean distance matrix was generated via vegdist() function, and distance-to-centroid values were calculated using betadisp(). Because group distances were normally distributed, differences in dispersion between cohorts, comparing NY (0, 12, or 24 M), PY, and HRY against healthy controls, were evaluated via a parametric, one-way Welch’s ANOVA with post-hoc Dunnett’s T3 test in GraphPad Prism (v.10.6.1).

### Reporting summary

Further information on research design is available in the Nature Portfolio Reporting Summary linked to this article.

## Supplementary information

Supplementary Information

Reporting Summary

Transparent Peer Review file

## Data Availability

Source data used for generation of figures are provided with this paper. The cytokine and inflammatory marker data generated in this study have been deposited in the Dryad repository and can be accessed at https://doi.org/10.5061/dryad.612jm64k4. Clinical data from the cohort that support the findings of this study are available under restricted access due to the sensitivity of information and patient confidentiality. Access to processed deidentified data may be available upon reasonable request to the corresponding authors. A reasonable request entails sharing of de-identified data within a specific scope tied to the publication, with the intent to replicate or verify published findings, respecting ethical guidelines including patient confidentiality, intellectual property and institutional privacy restrictions, with data delivered in the original format it was stored or analyzed. Reasonable requests for data sharing will be addressed within 10 business days. Data are located in a controlled access electronic storage managed by the University of California Los Angeles Health System.
