# Supplementary material for: Distinct inflammatory marker profiles of adolescents and young adults among the HIV risk, infection, and treatment spectrum
Source: Nat Commun. 2026 Sep 25;17:11266. doi: 10.1038/s41467-026-78034-6 (PMC13720089; doi:10.1038/s41467-026-78034-6)
Supplement: Supplementary file 1 [file 41467_2026_78034_MOESM1_ESM.pdf]

Supplemental Table 1. Proportion of Newly Diagnosed Youth (NY) with sexually transmitted infections during each study visit

	NY 0M		NY 12M		NY 24M		p
	N=93		N=63		N=56		
**STI at blood draw**							
**Gonorrhea**	14	15.1%	5	7.9%	5	8.9%	0.31
**Chlamydia**	10	10.8%	5	7.9%	5	8.9%	0.83
**Syphilis**	22	23.7%	16	25.4%	9	16.1%	0.42
**Any**	38	40.9%	22	34.9%	15	26.8%	0.22

Supplemental Table 2. Plasma immune-related mediators and receptors profiled via proteomics

	Protein	UniProt ID	Protein Definition	Function, Marker, and Disease Context
1	CD163	Q86VB7	Cluster of differentiation encoded by gene 163	Immune activation on surface of monocytes; upregulation in macrophage activation, disease progression.
2	D-dimer	N/A	Protein fragment released by clot breakdown	Marker of inflammation, involved in clot formation and breakdown; marker of disease progression, mortality in HIV.
3	CD257	Q9Y275	B-cell activating factor	Activation and proliferation of B cells / regulates B-cell survival, marker of immune activation.
4	CXCL13	O43927	Chemokine ligand encoded by CXCL13 gene	Chemotaxis of B cells, upregulated with inflammation.
5	PLA2	P04054	Phospholipase A2	Regulates inflammatory responses; mediates oxidative stress and increases brain inflammatory responses.
6	CXCL9	Q07325	Chemokine C-X-C motif ligand 9	Monokine induced by gamma interferon; chemotaxis of B cells, T cell recruitment, marker of immune activation, role in neuroinflammation.
7	VCAM-1	P19320	Vascular Cell Adhesion Molecule 1	Immune activation and inflammation; endothelial dysfunction; neurocognitive impairment.
8	ICAM-1	P05362	Intercellular Adhesion Molecule 1	CD54; cell surface adhesion in endothelial, immune cells, leukocytes; chronic immune activation; HIV replication.
9	CD120b	P20333	Tumor necrosis factor receptor II	Receptor on CD4s and other immune/ non-immune cells; marker of inflammation; increased in HIV viremia, influences T cell survival.
10	CD120a	P19438	Tumor necrosis factor receptor I	Major receptor for TNF-alpha. Activates transcription factor NF-κB, mediates apoptosis, regulates inflammation.
11	CD27	P26842	Cluster of differentiation 27	Part of TNF-receptor superfamily. Memory B cell marker; immune checkpoint molecule; immune activation downregulates CD27; reservoir for HIV.
12	EGF	P01133	Epidermal growth factor	Role in control of tumor growth and progression, apoptosis, and angiogenesis, when dysregulated can lead to tumor formation.
13	FGF-2	P09038	Fibroblast growth factor 2	Role in embryonic/ stem cell development, growth, repair, wound healing, secreted by adipocytes; dysregulated in cancer progression
14	CCL11	P51671	C-C motif chemokine ligand 11	Binds to CCR3 receptor, induces allergic reactions.
15	TGF-α	P01135	Transforming growth factor alpha	Produced in macrophages, brain cells, keratinocytes; cell proliferation, neural regeneration; dysregulation in angiogenesis, tumor proliferation.
16	G-CSF	P09919	Granulocyte colony stimulating factor	Granulocyte production; dysregulation enhances tumors.
17	FLT3L	P49771	FMS-like tyrosine kinase 3 ligand	Growth factor of immune and dendritic cells; reduces IL-6 and TNF; helps form TREG cells, anti-inflammatory activity.
18	GM-CSF	P04141	Granulocyte-Macrophage Colony-stimulating factor	Granulocyte production as in G-CSF but broader; enhances macrophage and eosinophil proliferation.
19	CX3CL1	P78423	C-X3-C motif chemokine ligand 1	Present in neural and endothelial cells; necessary for microglial cell migration; upregulated in brain or kidney inflammation.
20	IFN-α2	P48551	Interferon alpha 2	Antiviral activity, inhibits cell proliferation, regulates immune system activation; decreases tumor formation; is still pro-inflammatory cytokine.
21	IFN-γ	P01579	Interferon gamma	Activates T-cells, NK cells, NKT cells; Pro-inflammation, innate, adaptive immunity anti-viral effect.
22	CXCL1	P09341	C-X-C motif chemokine ligand 1	Chemoattractant for neutrophils and non-hematopoietic cells to sites of injury or infection; regulates immune/ inflammatory responses.
23	IL-10	P22301	Interleukin 10	Anti-inflammatory cytokine, inhibition of pro-inflammatory cytokines.
24	CCL7	P80098	C-C motif chemokine ligand 7	Recruits leukocytes to infected tissues to mediate immune response; dysregulation enhances HIV disease progression.
25	IL12p40	P29460	Interleukin 12 p40 / IL-12B	Natural killer cell stimulatory factor 2; subunit of interleukins 12 and 23. Involved in allergic and autoimmune diseases.
26	CCL22	O00626	C-C motif chemokine ligand 22	Dysregulation enhances tumor replication.
27	IL12p70	N/A	Interleukin 12p70	Induces NK cells, proliferates Th1 cells by IFN-γ production; augments cytotoxicity of CD8+ and NK cells; enhances adhesion molecule expression.
28	IL-13	P35225	Interleukin 13	Induction of reactive airway disease, also has anti-inflammatory properties; similar to IL-4.
29	IL-15	P40933	Interleukin 15	Inflammatory cytokine similar to IL-2; induces proliferation of NK cells.
30	CD40-L	P29965	Soluble form of CD40L	Member of TNF family; expressed in a variety of cell types, including B cells, has proinflammatory and procoagulant effects.
31	IL-17A	Q16552	Interleukin 17A	Pro-inflammatory cytokine produced by activated T cells; stimulates expression of COX-2 and IL-6; enhances nitric oxide.
32	IL-1RA	P18510	IL-1 receptor antagonist protein	Natural inhibitor of the pro-inflammatory effect of IL1β.
33	IL-1α	P01583	Interleukin 1a	Activated with inflammation, fever and sepsis.
34	IL-9	Q01113	Interleukin 9	Role in cell proliferation; prevents apoptosis; part of immune responses against parasites; role in allergic respiratory diseases.
35	IL-1β	P01584	Interleukin 1b	Leukocyte pyrogen, role in inflammatory responses, auto inflammatory diseases, gut inflammation in HIV, depletion of T cells
36	IL-2	P60568	Interleukin 2	Originates from activated CD4+ CD8+ T cells. Stimulates growth of helper, cytotoxic regulatory T cells; downregulation enhances inflammation.
37	IL-3	P08700	Interleukin 3	Produced by activated T cells to enhance pathogen clearance; induces inflammation; stimulates stem cells to become myeloid progenitor cells.
38	IL-4	P05112	Interleukin 4	Produced by TH cells; Anti-inflammation, T-cell and B-cell proliferation, B-cell differentiation.
39	IL-5	P05113	Interleukin 5	Stimulates B cell growth and increases immunoglobulin secretion - primarily IgA; key mediator in eosinophil activation; role in asthma/ allergies.
40	IL-6	P05231	Interleukin 6	Proinflammatory cytokine, produced in activated leukocytes; triggers production of C-Reactive protein. Associated with HIV viremia.
41	IL-7	P13232	Interleukin 7	Differentiates stem cells into lymphoid progenitor cells; role in B/ T cell development; high levels disrupt T cell development w/ B cell development.
42	IL-8	P10145	Interleukin 8	Pro-inflammation, chemotaxis, angiogenesis
43	CXCL10	P02778	C-X-C motif chemokine ligand 10	Chemoattractant for activated T cells, monocytes, dendritic cells and NK cells, guides them to sites of infection/ inflammation.
44	CCL2	P13500	C-C motif chemokine ligand 2	Chemokine ligand 2 (CCL2); Induces monocytic infiltrates, particularly in kidneys; role in neuroinflammation.
45	CCL3	P10147	C-C motif chemokine ligand 3	Produced by macrophages and monocytes when stimulated by IL-1β.
46	CCL4	P13236	C-C motif chemokine ligand 4	Induces chemotaxis/triggers release of bioactive molecules; affects monocytes, T lymphocytes, dendritic cells, NK cells, platelets.
47	TNF-α	P01375	Tumor Necrosis Factor alpha	TNF, cachexin, or cachectin; TNF-α: Regulates immune cells; endogenous pyrogen; role in apoptotic cell death, inflammation, sepsis.
48	TNF-β	P01374	Tumor Necrosis Factor beta	Lymphotoxin-alpha (LT-α): anti-proliferative activity. Role in immune regulation. Unregulated expression induces tumor formation.
49	VEGF	P15692	Vascular Endothelial Growth factor	Stimulates formation of blood vessels; present following brain injury; activated by TNF response; upregulated in inflammatory conditions.
