# Supplementary material for: Distinct inflammatory marker profiles of adolescents and young adults among the HIV risk, infection, and treatment spectrum
Source: Nat Commun. 2026 Sep 25;17:11266. doi: 10.1038/s41467-026-78034-6 (PMC13720089; doi:10.1038/s41467-026-78034-6)
Supplement: Supplementary file 2 [file 41467_2026_78034_MOESM2_ESM.pdf]

nature portfolio

Corresponding author(s): NCOMMS-25-97667C

Last updated by author(s): Sep 2, 2026

Reporting Summary

Nature Portfolio wishes to improve the reproducibility of the work that we publish. This form provides structure for consistency and transparency in reporting. For further information on Nature Portfolio policies, see our Editorial Policies and the Editorial Policy Checklist.

Statistics

For all statistical analyses, confirm that the following items are present in the figure legend, table legend, main text, or Methods section.

n/a	Confirmed	
☐	☒	The exact sample size (*n*) for each experimental group/condition, given as a discrete number and unit of measurement
☐	☒	A statement on whether measurements were taken from distinct samples or whether the same sample was measured repeatedly
☐	☒	The statistical test(s) used AND whether they are one- or two-sided<br>*Only common tests should be described solely by name; describe more complex techniques in the Methods section.*
☐	☒	A description of all covariates tested
☒	☐	A description of any assumptions or corrections, such as tests of normality and adjustment for multiple comparisons
☐	☒	A full description of the statistical parameters including central tendency (e.g. means) or other basic estimates (e.g. regression coefficient) AND variation (e.g. standard deviation) or associated estimates of uncertainty (e.g. confidence intervals)
☐	☒	For null hypothesis testing, the test statistic (e.g. *F*, *t*, *r*) with confidence intervals, effect sizes, degrees of freedom and *P* value noted<br>*Give P values as exact values whenever suitable.*
☒	☐	For Bayesian analysis, information on the choice of priors and Markov chain Monte Carlo settings
☒	☐	For hierarchical and complex designs, identification of the appropriate level for tests and full reporting of outcomes
☐	☒	Estimates of effect sizes (e.g. Cohen's *d*, Pearson's *r*), indicating how they were calculated

Our web collection on statistics for biologists contains articles on many of the points above.

Software and code

Policy information about availability of computer code

Data collection	Data collection for demographics and potential covariates was achieved from trained interviewers using Android tablets. Interviewers enter the participants' responses in the CommCare mobile-Web electronic assessment and case management system that is cloud based and Health Insurance Portability and Accountability Act (HIPAA) compliant, by Dimagi Inc.
Data analysis	Stastical data was analyzed using R (R version 4.4.2 Copyright (C) 2024 The R Foundation for Statistical Computing). R packages used were dplyr and psych. Proteomic assessment was conducted using and online platform Enricher, GraphPad Prism, and Qiagen Ingenuity Pathway Analysis.

For manuscripts utilizing custom algorithms or software that are central to the research but not yet described in published literature, software must be made available to editors and reviewers. We strongly encourage code deposition in a community repository (e.g. GitHub). See the Nature Portfolio guidelines for submitting code & software for further information.

Data

Policy information about availability of data

All manuscripts must include a data availability statement. This statement should provide the following information, where applicable:

- Accession codes, unique identifiers, or web links for publicly available datasets
- A description of any restrictions on data availability
- For clinical datasets or third party data, please ensure that the statement adheres to our policy

Data availability
Source data used for generation of figures are provided with this paper. The cytokine and inflammatory marker data generated in this study have been deposited in the Dryad repository and can be accessed at DOI: https://doi.org/10.5061/dryad.612jm64k4. Clinical data from the cohort that support the findings of this study are available under restricted access due to the sensitivity of information and patient confidentiality. Access to processed deidentified data may be available upon reasonable request to the corresponding authors. Data are located in a controlled access electronic storage managed by the University of California Los Angeles Health System.

Research involving human participants, their data, or biological material

Policy information about studies with human participants or human data. See also policy information about sex, gender (identity/presentation), and sexual orientation and race, ethnicity and racism.

Reporting on sex and gender	All participants in our study self-identified as male. Due to the recruitment procedures, most identified as gay.
Reporting on race, ethnicity, or other socially relevant groupings	We acknowledge that race is a social construct and categorizations may not adequately reflect an indivdual's identity. Participants self reported their race and ethnicity. We categorized race and ethnicity in the following way, to be consistent with other papers from the ATN CARES study. Black was self reported Black, African-American, or Hispanic-Black. White was considered as non-Hispanic White. Asian, Native American, Alaska Native, and Pacific Islander were collapsed into a single group due to small numbers. Hispanic was considered anyone who marked Hispanic and did not fall into one of the previous categories.
Population characteristics	Youth aged 12-24 were enrolled in the CARES (Comprehensive Adolescent Recruitment and Engagement Strategies) study, sponsored by the NIH Adolescent Trials Network (ATN), U19 grant (U19HD089886), which recruited youth at the highest risk of acquiring HIV, including gay, bisexual, transgender, and homeless youth, and youth living with HIV in the Los Angeles and New Orleans areas. Youth enrolled in ATN studies in the U.S. are representative of young individuals living with or at risk for HIV acquisition across the country
Recruitment	Participants were recruited and screened from gay-identified community-based organizations, homeless shelters, adolescent medical clinics, as well as social media, dating apps, and referrals. Participating ATN147 study sites include David Geffen School of Medicine at the University of California at Los Angeles, Department of Pediatrics, Division of Infectious Diseases, the Los Angeles lesbian, gay, bisexual, and transgender (LGBT) Center, and Tulane University School of Medicine, Department of Adolescent Medicine. Along with screening for participants without HIV infection, participants who tested positive for HIV were recruited through referral from the ATN149 CARES high-risk cohort without HIV and direct referrals from the community. Control participants were recruited from participants in a study of first-line responders during the COVID-19 pandemic conducted at UCLA. They were all male individuals, in good health, without HIV infection or concurrent STIs, who worked as frontline workers (e.g., firefighters, physicians, nurses) in Los Angeles, CA between 2020 and 2022, aged 20 to 27 years of age.
Ethics oversight	All procedures were approved by the University of California, Los Angeles institutional review board and the Adolescent Medicine Trials Network (ATN) Study Monitoring Committee. The study was carried out in accordance with the declaration of Helsinki, and informed consent was obtained from patients at enrollment .

Note that full information on the approval of the study protocol must also be provided in the manuscript.

Field-specific reporting

Please select the one below that is the best fit for your research. If you are not sure, read the appropriate sections before making your selection.

☒ Life sciences ☐ Behavioural & social sciences ☐ Ecological, evolutionary & environmental sciences

For a reference copy of the document with all sections, see nature.com/documents/nr-reporting-summary-flat.pdf

Life sciences study design

All studies must disclose on these points even when the disclosure is negative.

Sample size	From the parent grant ATN CARES, we selected participants who had additional blood samples stored. This included 262 participants, with 93 from ATN 147, 24 from ATN 148, 110 from ATN 149, and 35 controls participants.
Data exclusions	Exclusion criteria included (1) active drug or alcohol use or dependence that, in the opinion of the site investigator, would interfere with adherence to study requirements, (2) any acute, chronic, or recent and clinically significant medical condition that, in the opinion of the site

	investigator, would interfere with adherence to study requirements or jeopardize the safety or rights of the participant, (3) chronic or recurrent use of medications that modify host immune response, for example, oral or parenteral steroids and cancer chemotherapy, and (4), being over the age of 24 or younger than 12 years. Additional individual inclusion or exclusion criteria specific to the focus of each ATN study (147, 148, or 149) were present. For ATN 147, all baseline specimens were included, but a detectable virema at 12 months or later was excluded from the proteomic analysis. Similarly, participants from the 148 study that had a detectable virema were also excluded.
Replication	This was an observational study. The data processing code has be rerun multiple times by different people to verify results.
Randomization	This was an observational study, so there was no randomization. Potential confounding factors were assessed by a detailed questionnaire regarding demographic factors such as age, race/ethnicity, gender, and sexual orientation, plus behavioral elements including sexual history and practices, drug and alcohol use. These were considered for adjustment in our model. Additionally, By including in the data analysis only participants who obtained an undetectable plasma viral load following ART initiation in ATN 147 and 148, we aimed to exclude participants who might have ongoing inflammation triggered by HIV viremia.
Blinding	This was an observational study and participants were not blinded into groups

Reporting for specific materials, systems and methods

We require information from authors about some types of materials, experimental systems and methods used in many studies. Here, indicate whether each material, system or method listed is relevant to your study. If you are not sure if a list item applies to your research, read the appropriate section before selecting a response.

Materials & experimental systems

n/a	Involved in the study
☒	☐ Antibodies
☒	☐ Eukaryotic cell lines
☒	☐ Palaeontology and archaeology
☒	☐ Animals and other organisms
☒	☐ Clinical data
☒	☐ Dual use research of concern
☒	☐ Plants

Methods

n/a	Involved in the study
☒	☐ ChIP-seq
☒	☐ Flow cytometry
☒	☐ MRI-based neuroimaging

Plants

Seed stocks	*Report on the source of all seed stocks or other plant material used. If applicable, state the seed stock centre and catalogue number. If plant specimens were collected from the field, describe the collection location, date and sampling procedures.*
Novel plant genotypes	*Describe the methods by which all novel plant genotypes were produced. This includes those generated by transgenic approaches, gene editing, chemical/radiation-based mutagenesis and hybridization. For transgenic lines, describe the transformation method, the number of independent lines analyzed and the generation upon which experiments were performed. For gene-edited lines, describe the editor used, the endogenous sequence targeted for editing, the targeting guide RNA sequence (if applicable) and how the editor was applied.*
Authentication	*Describe any authentication procedures for each seed stock used or novel genotype generated. Describe any experiments used to assess the effect of a mutation and, where applicable, how potential secondary effects (e.g. second site T-DNA insertions, mosiacism, off-target gene editing) were examined.*
