# Supplementary material for: Distinct inflammatory marker profiles of adolescents and young adults among the HIV risk, infection, and treatment spectrum
Source: Nat Commun. 2026 Sep 25;17:11266. doi: 10.1038/s41467-026-78034-6 (PMC13720089; doi:10.1038/s41467-026-78034-6)
Supplement: Supplementary file 3 [file 41467_2026_78034_MOESM3_ESM.pdf]

nature portfolio

Peer Review File

Distinct Inflammatory Marker Profiles of Adolescents and Young Adults among the HIV risk, infection, and treatment spectrum

Corresponding Author: Professor Karin Nielsen-Saines

This file contains all reviewer reports in order by version, followed by all author rebuttals in order by version.

Version 0:

Reviewer comments:

Reviewer #1

(Remarks to the Author)
The manuscript from Dong, et al. describes a detailed plasma proteomic profiling analysis in a cohort of 262 adolescents and young adults, including healthy controls (HC), high-risk youth (HRY), newly diagnosed HIV-positive youth (NY), and previously diagnosed youth on long-term ART (PY). The authors identify distinct but partially overlapping immune signatures across study groups. Newly diagnosed youth exhibited the most pronounced immune perturbations at baseline, while high-risk youth showed broad immune heterogeneity. Long-term ART was associated with convergence toward healthy immune profiles, although residual immune alterations persisted.
The authors emphasized that, compared to HC, HRY display systemic immune dysregulation involving several microbial sensing and inflammatory pathways. However, these observed differences between HRY and healthy controls may be confounded by the high prevalence of sexually transmitted infections (STIs) within the HRY group (24.6%). Given that the study includes 110 HRY participants, the cohort seems sufficiently powered to stratify HRY into two subgroups with and without STIs. An analysis comparing HRY without STIs to healthy controls would be particularly informative in clarifying immune signatures attributable to HIV exposure risk rather than concurrent infection.
Similarly, among the 93 participants in the NY group, nearly half (49.6%) had concurrent STIs. Again, Stratified analyses comparing NY participants with vs. without STIs – both at baseline and in response to ART at 12 and 24 months – would strengthen interpretation of immune changes attributed specifically to acute HIV infection and ART-mediated immune reconstitution. In addition, data shown in Figure 3A/D/F suggest a relatively slow resolution of immune activation following ART initiation in newly diagnosed HIV infection, underscoring the potential importance of long-term ART and concurrent treatment of STIs in clinical care of adolescents and young adults at high risk or living with HIV infection.
Overall, due to potential confounding factors – including differences in STIs prevalence and Race/Ethnicity between high-risk or HIV infected cohorts compared to healthy control cohort in the study - it remains inconclusive whether the elevated markers observed in HRY and HIV infected cohorts represent immune signatures specific to HIV exposure or infection per se, rather than reflecting broader inflammatory or sociodemographic influences.

Reviewer #2

(Remarks to the Author)
In this manuscript, Huan Vinh Dong and colleagues investigate the plasma proteomic profiles of young people living with HIV, individuals at high risk of HIV acquisition (HRY), and healthy controls. The topic is highly relevant, as adolescents and young adults are frequently underrepresented in HIV research despite accounting for a substantial proportion of new HIV infections worldwide. A better understanding of the immunological and proteomic landscape associated with HIV acquisition in this key population could indeed inform future social and biomedical prevention strategies.
The cohorts are relatively large and appear, at first glance, to be reasonably well defined, and the overall proteomic approach is appropriate. However, the manuscript suffers from substantial problems in writing quality, methodological clarity, data presentation, and interpretation. In its current form, the study does not convincingly support many of the authors’ claims.
General comments
Language and terminology: given that this paper focuses on vulnerable populations, the authors should adhere to UNAIDS-recommended, person-first, non-stigmatizing language. Several terms used throughout the manuscript are outdated or

inappropriate, including “HIV patients,” “HIV-infected children,” and “HIV disease.” These expressions should be consistently revised.
Figure quality and consistency
a. Several figures are of poor graphical quality, making them difficult to interpret.
b. Figure legends are inconsistent across the manuscript. For example, healthy controls are referred to as “HC” in some figures and “Healthy” in others. This must be standardized.
c. Color coding is also inconsistent. In Figure 3, the same groups are represented by different colors across subpanels, which is confusing and unacceptable.
Methodological clarity
a. It is unclear whether the proteomic values obtained from the two analytical platforms represent absolute concentrations or relative expression levels.
b. The methodology for Gene Ontology analysis is poorly described. It is not clear whether this analysis was performed directly through IPA or using an independent pipeline. This must be clarified.

Major concerns regarding the analysis and interpretation
• While groups are matched for age and compared for ethnicity and STI status at blood draw, several critical confounding factors relevant to inflammatory and immunoregulatory proteomics are not addressed. In particular, obesity and smoking status are not included or discussed. Additionally, other exclusion criteria appear to rely on physician discretion but are not clearly specified in the Methods.
• In Figure 1B, the tight clustering of NY samples at 24 months is unexpected. One would anticipate these samples to move closer to healthy controls over time. Instead, they form a distinct cluster, raising concerns about potential technical bias. Were samples randomized during processing? Was batch effect assessed and corrected?
Furthermore, the description of Figure 1B in the text is overstated. The claim that NY 0M samples are more separated from HC than NY 12M is not convincingly supported by the PCA. Differences in ellipse size are subtle and do not justify the strong biological interpretation provided.
• In Figure 2C, it is unclear which group (HC or HRY) shows upregulation or downregulation of proteins. This information should be explicitly annotated in the figure. Adjusting the log2 fold-change scale would also substantially improve readability.
• In Figure 2F, it is unclear why only upregulated proteins were included in the network analysis. Downregulated proteins may be equally informative and should not be excluded without justification. Moreover, it is not clear whether the proteins shown correspond to those in Figure 2C, as the nomenclature differs.
The figure is difficult to interpret, and the accompanying text is vague and overstated. The sentence describing a “fragmented circuit topology” is unclear and unsupported. The authors should reconsider both the figure design and the biological interpretation. What exactly is meant by “fragmented circuit topology,” and how is this objectively defined?
• In Figure 3A, the authors claim that NY 0M is the most dysregulated group and that protein expression progressively normalizes at 12 and 24 months of ART. This conclusion is not supported by the data shown, as the three timepoints appear highly similar. A hierarchical clustering analysis of samples, colored by timepoint, would be far more appropriate to assess whether timepoints cluster separately or are intermixed. Such an analysis could reveal a persistent HIV-associated proteomic signature despite prolonged ART, which would be an important finding.
• In Figure 3C, the authors state that PCA shows persistent separation from HC at both 12M and 24M, indicating incomplete immune reconstitution. However, 24M appears to be the most distinct group, not intermediate. This again raises concerns similar to those mentioned in my second comment.
• Figure 3E further strengthens concerns about potential technical or analytical bias. Pathways are consistently upregulated at 24M, regardless of whether they are also enriched at 0M or 12M. This pattern is biologically puzzling.
If these pathways reflect acute infection–related inflammation, one would expect higher activation at 0M with normalization over time. Conversely, if they represent chronic inflammation under ART, one would expect low levels at 0M and progressive increases. The consistent maximal upregulation at 24M lacks interpretation and is not adequately discussed.
• In Figure 3F, it is unclear how upstream regulators were inferred. They appear to be proteins from the original dataset rather than results of a distinct upstream regulator analysis. This needs clarification. Including HC as a reference group would also strengthen this analysis.
• In Figure 3G, p-values are missing and should be provided.
• In Figure 4C, as in Figure 2C, the direction of regulation (which group shows upregulation or downregulation) should be clearly indicated to facilitate interpretation.
• In line 221, TNF is incorrectly described as an immunosuppressive mediator, whereas it is a well-established pro-inflammatory cytokine.
• The identification of 10 proteins downregulated in the HRY group is one of the most interesting findings in the manuscript, yet it is underdeveloped. These proteins are largely pro-inflammatory. This raises an important and intriguing hypothesis: could a lower inflammatory baseline contribute to protection from HIV acquisition? This aspect of the HRY group is insufficiently discussed and represents a missed opportunity.
• Figures 5C and 5D are of very poor graphical quality and use different color palettes. These figures should be reformatted for clarity and consistency.
• In Figures 5E–F, the discussion in the text is minimal and confusing. CXCL10 is mentioned in the text, but the figure displays CXCL9. Similar inconsistencies occur elsewhere. Additionally, CCL2 is discussed but not visible in the figure.

In summary, while the cohorts are valuable and the study has the potential to make a meaningful contribution to the field, the manuscript in its current form is not ready for publication. Substantial revisions are required, including improvements in writing, methodological transparency, figure quality, statistical rigor, and biological interpretation. Without these changes, the data remain underutilized and many conclusions are insufficiently supported.

Version 1:

Reviewer comments:

Reviewer #1

(Remarks to the Author)
The authors have adequately addressed my previous concerns, and the revised manuscript has significantly improved. I have no further comments.

Reviewer #2

(Remarks to the Author)
The authors have clearly engaged with my prior comments and made several constructive improvements (including figure corrections/updates and additional stratified analyses), which I appreciate. However, despite these revisions, the overall conclusions remain unconvincing because key analytical steps and quality-control measures are still insufficiently described, and the manuscript continues to draw mechanistic inferences that are not adequately supported by the presented data. The central claims (e.g., “blunted innate sensing,” “suppressed type I IFN signaling,” “NF-κB/STAT3 activation,” “immune exhaustion/priming,” cGAS–STING involvement) remain unsupported by orthogonal cellular or functional data as they are based on inference from measurement of 49 plasma proteins. As written, the study is best framed as descriptive proteomic profiling, not mechanistic immunology.
Major comments
1) Data processing and normalization
The Methods state that proteomic data were “log-transformed and normalized prior to analysis,” and that PCA was used to visualize global patterns. However, the manuscript does not specify what normalization approach was applied (e.g., median/quantile/plate-wise, internal standards, bridging controls), whether batch effects were formally evaluated, or whether any batch correction was applied (and if so, using which method and covariates). Given that PCA/clustering are central to the narrative, and the authors corrected an error in the PCA figure, it is important to demonstrate that the observed structure is not driven by processing batches, plate effects, site, or run date.
Please provide:
1. a clear description of normalization and any batch correction method used
2. evidence that batch effects were assessed (e.g., PCA colored before and after correction)
2) Mechanistic claims from IPA based on plasma proteins are overreaching
The manuscript continues to infer “upstream regulator activation/inhibition” (e.g., NF-κB, STAT3, type I interferon) and pathway status (e.g., cGAS–STING) from directional changes in a limited circulating protein panel using Ingenuity Pathway Analysis (IPA).
This is a methodologically precarious leap. Plasma cytokine/chemokine concentrations reflect a net outcome of secretion, compartmentalization, receptor shedding, clearance kinetics, binding proteins, and consumption and not intracellular transcription factor activity. Consequently, claims of “activation” or “inhibition” of master regulators based on extracellular concentrations alone are over-interpretations. What orthogonal evidence supports the predicted upstream regulator states, beyond IPA extrapolation from 49 circulating proteins?
To claim mechanistic, the manuscript would require at least the following revisions:
• Reframe IPA outputs explicitly as hypothesis-generating rather than mechanistic conclusions.
• Remove or substantially soften language implying demonstrated intracellular signaling states (e.g., “inhibition of type I interferon signaling,” “NF-κB activation,” “blunted innate sensing,” “cGAS–STING activation”) unless validated with orthogonal assays.
I would suggest also some mechanistical validations to strength the hypothesis and observations, such as: (a) PBMC transcriptomics (bulk or single-cell) or targeted qPCR (to check for IFN stimulated genes, NF-kB, etc) (b) intracellular signaling readouts (phospho-flow / CyTOF), e.g., pSTAT1/2 (type I IFN), pSTAT3, p-p65 (NF-κB).
3) “Immune exhaustion” and “immune priming”
The manuscript links elevated soluble checkpoint-related markers (notably soluble PD-L1) to “immune exhaustion,” and interprets the HRY profile as “primed/maladaptively primed.” However, no functional assays are presented to support exhaustion, dysfunction, or altered responsiveness.
Soluble checkpoint molecules are not sufficient proxies for T cell exhaustion. Exhaustion is a cell-state defined by single-cell phenotype and functon(co-inhibitory receptor co-expression, transcriptional state, impaired proliferation/cytokine production, etc.), not by plasma marker levels alone.
They should replace “exhaustion” language with conservative phrasing (e.g., “increased soluble immune checkpoint markers” or “checkpoint-associated signature”) unless functional data are added.
Clearly distinguish correlation from mechanism throughout the Discussion.
Similarly, functional data would be required to demonstrate real exhaustion of HRY Tcells: proliferation assay (CFSE/CTV), cytokine production after stimulation (anti-CD3/CD28; PMA/ionomycin) and cytotoxic markers along with a flow characterization of exhaustion markers (PD-1, TIGIT, LAG-3, TIM-3, CD39, TOX) plus memory/activation context.
4) STI confounding: stratification helps but is still not fully convincing for the strongest claims
The authors added STI-stratified analyses (response document pp. 1–2), concluding STI status “did not significantly alter general immune profiles.” This is helpful, but the interpretation remains too definitive.
For the key conclusions (e.g., “blunted IFN,” “primed state”), re-run and show results restricted to STI-negative participants only and indicate whether the same IPA calls persist with comparable effect sizes. Do the signature proteins and IPA predictions remain when analyses are strictly restricted to STI-negative participants, and do they survive correction with

adequate power?
Minor / specific comments
1. Figure 1B: The spread/dispersion of groups should be discussed with respect to cohort heterogeneity. Could the wider dispersion in some groups reflect unequal STI burden or other measured covariates? Please explicitly test whether dispersion correlates with STI status or other available metadata.
2. Figure 2 (HRY profiling): Some upregulated markers are in the β-chemokine family (e.g., CCR5 ligands) which can competitively inhibit HIV entry. This alternative biological interpretation (potential protective/competitive mechanism rather than "blunted sensing") should be discussed.
3. Terminology mismatch (HRY vs HIV infection): In the description of Fig. 2E/related text, the phrase "inflammatory network in HIV infection" is inaccurate if referring to HRY who are HIV-negative. Please correct to avoid conceptual confusion.
4. IPA general limitation: Please add explicit limitation language: IPA upstream regulator analysis is not direct evidence of intracellular activation/inhibition, especially when input is limited to circulating proteins.
5. No validation/perturbation: The manuscript should not read as if mechanisms were demonstrated. If the authors cannot add functional experiments, the Discussion should be rewritten to clearly present hypotheses and avoid therapeutic suggestions that rely on unproven mechanistic states.
6. Nomenclature consistency: Ensure consistent labeling between text and figures (e.g., eotaxin vs CCL11; GRO1 vs CXCL1, etc.). This inconsistency was raised before at other figures in my first review, but those specific molecules of figure 5 were correctly labeled in the previous version.
7. Psychosocial stressors: The Discussion mentions psychosocial stressors as drivers (your noted lines ~314–315). If authors invoke this, they should either (i) analyze available stress-related metadata, or (ii) clearly label this as speculation and expand the confounding discussion. [Gregor MF, Hotamisligil GS. Inflammatory mechanisms in obesity. Annu Rev Immunol (2011); Arnson Y, Shoenfeld Y, Amital H. Effects of tobacco smoke on immunity, inflammation and autoimmunity. J Autoimmun (2010).

Open Access This Peer Review File is licensed under a Creative Commons Attribution 4.0 International License, which permits use, sharing, adaptation, distribution and reproduction in any medium or format, as long as you give appropriate credit to the original author(s) and the source, provide a link to the Creative Commons license, and indicate if changes were made.
In cases where reviewers are anonymous, credit should be given to 'Anonymous Referee' and the source.
The images or other third party material in this Peer Review File are included in the article's Creative Commons license, unless indicated otherwise in a credit line to the material. If material is not included in the article's Creative Commons license and your intended use is not permitted by statutory regulation or exceeds the permitted use, you will need to obtain permission directly from the copyright holder.
To view a copy of this license, visit https://creativecommons.org/licenses/by/4.0/

Response to reviewers: NCOMMS-25-97667-T

Reviewer #1:

The manuscript from Dong, et al. describes a detailed plasma proteomic profiling analysis in a cohort of 262 adolescents and young adults, including healthy controls (HC), high-risk youth (HRY), newly diagnosed HIV-positive youth (NY), and previously diagnosed youth on long-term ART (PY). The authors identify distinct but partially overlapping immune signatures across study groups. Newly diagnosed youth exhibited the most pronounced immune perturbations at baseline, while high-risk youth showed broad immune heterogeneity. Long-term ART was associated with convergence toward healthy immune profiles, although residual immune alterations persisted.

Response: We thank the reviewer for the positive and constructive feedback which has enabled us to improve our manuscript significantly through this revision. We have made a genuine attempt to thoroughly address all concerns that the reviewer raised, as described below.

1) The authors emphasized that, compared to HC, HRY display systemic immune dysregulation involving several microbial sensing and inflammatory pathways. However, these observed differences between HRY and healthy controls may be confounded by the high prevalence of sexually transmitted infections (STIs) within the HRY group (24.6%). Given that the study includes 110 HRY participants, the cohort seems sufficiently powered to stratify HRY into two subgroups with and without STIs. An analysis comparing HRY without STIs to healthy controls would be particularly informative in clarifying immune signatures attributable to HIV exposure risk rather than concurrent infection.

   Response: We thank the reviewer for the thoughtful comment. In the HRY cohort, we had 23 individuals (21%) who had any of the three STIs evaluated for, and 87 individuals who did not have STIs. As requested by the reviewer, we have performed additional analyses to compare the immune profiles of (i) HRY without STIs (N=84) and (ii) HRY with STIs (N=23) to healthy controls. We found that the status of STIs did not significantly alter the general immune profiles of HRY patients, with only 4 proteins out of 30 significantly affected proteins that were specifically affected in the HRY-(+)STIs group. We have now included these findings in **Figs. 2G-2I and Supplementary Figure 1** of the revised manuscript.

2) Similarly, among the 93 participants in the NY group, nearly half (49.6%) had concurrent STIs. Again, Stratified analyses comparing NY participants with vs. without STIs – both at baseline and in response to ART at 12 and 24 months – would strengthen interpretation of immune changes attributed specifically to acute HIV infection and ART-mediated immune reconstitution.

   Response: We thank the reviewer for the thoughtful comment. The newly diagnosed cohort (NY) is distinct from the other cohorts in that longitudinal samples were collected prospectively at 3 timepoints. Cytokine profiles were evaluated at baseline (prior to start of ARTs), 12 months and 24 months post ART initiation, allowing cytokine trajectories over time. We have now included a supplemental table **(Supplementary Table 1)** detailing the frequency of concurrent STIs at each timepoint. The reviewer is correct in that there was a high prevalence of STIs across all visits (41%, 25%, and 22%) at each respective timepoint. Because participants could present with more than one infection, STI categories were not mutually exclusive. We have therefore added an additional row to both **Table 1** and the **Supplementary Table 1** highlighting the number of unique participants with at least one STI at each timepoint.

   Additionally, we have now performed a stratified analysis comparing the proteomics of participants with and without STIs at baseline and subsequent timepoints to each other and to healthy controls.

Results demonstrate that the status of STIs did not alter the general immune profiles of NY patients at 12M and 24M, and with only 3 proteins out of 31 significantly affected proteins that were specifically affected in the NY:0M-(+)STIs group. We have now included these findings in **new Figs. 3J-3K** of the revised manuscript.

3) In addition, data shown in Figure 3A/D/F suggest a relatively slow resolution of immune activation following ART initiation in newly diagnosed HIV infection, underscoring the potential importance of long-term ART and concurrent treatment of STIs in clinical care of adolescents and young adults at high risk or living with HIV infection.

Response: We agree with the reviewer and have included this comment in the third paragraph of the discussion.

4) Overall, due to potential confounding factors – including differences in STIs prevalence and Race/Ethnicity between high-risk or HIV infected cohorts compared to healthy control cohort in the study - it remains inconclusive whether the elevated markers observed in HRY and HIV infected cohorts represent immune signatures specific to HIV exposure or infection per se, rather than reflecting broader inflammatory or sociodemographic influences.

Response: We thank the reviewer for raising this important point, which prompted additional analyses to address potential confounding by STI prevalence and demographic differences. As suggested, we performed additional stratified analyses on HRY and NY groups based on their STIs status at each study visit. These analyses showed largely comparable immune profiles between participants with and without STIs, indicating that overall immune signatures were specific to HIV exposure/infection and minimally affected by (+)STIs status. These new analyses are now included as **new Figs. 2G-2I**, **3J-3K,** and **Supplementary Figure 1** of the revised manuscript.

Since we have now performed stratified analyses with and without STIs within the cohort of newly diagnosed youth, high-risk youth, and healthy controls, we believe the inflammatory marker profiles reported are representative of HIV exposure. With respect to race and ethnicity, a large proportion of participants within each cohort were of Hispanic ethnicity, including the control group, reflecting the demographics of the population living in Los Angeles and the local HIV epidemic. We acknowledge that proportionally there was a higher proportion of white participants in the control group, nevertheless the demographic composition and ethnic background of ATN study participants and within the cohorts were similar and consistent with the demographics and epidemiology of HIV in youth (DeMonte et al, reference 25 main manuscript), supporting the comparability of the study groups.

Reviewer #2:

In this manuscript, Huan Vinh Dong and colleagues investigate the plasma proteomic profiles of young people living with HIV, individuals at high risk of HIV acquisition (HRY), and healthy controls. The topic is highly relevant, as adolescents and young adults are frequently underrepresented in HIV research despite accounting for a substantial proportion of new HIV infections worldwide. A better understanding of the immunological and proteomic landscape associated with HIV acquisition in this key population could indeed inform future social and biomedical prevention strategies.

The cohorts are relatively large and appear, at first glance, to be reasonably well defined, and the overall proteomic approach is appropriate. However, the manuscript suffers from substantial problems in writing quality, methodological clarity, data presentation, and interpretation. In its current form, the study does not convincingly support many of the authors' claims.

Response: We thank the reviewer for the careful evaluation of our manuscript and for highlighting the importance of studying adolescents and young adults in the context of HIV infection. In response, we have undertaken a comprehensive revision and made a genuine attempt to improve clarity and organization, expand methodological descriptions, refine data presentation, and revise the text ensuring that all interpretations are appropriately supported by data. In addition, we incorporated new analyses, including stratified assessments to strengthen the robustness of our findings. We hope that the revisions have substantially improved the transparency, rigor, and interpretability of the work, and more clearly convey the strength of the evidence.

General comments:

1) Language and terminology: given that this paper focuses on vulnerable populations, the authors should adhere to UNAIDS-recommended, person-first, non-stigmatizing language. Several terms used throughout the manuscript are outdated or inappropriate, including "HIV patients," "HIV-infected children," and "HIV disease." These expressions should be consistently revised.

   Response: We thank the reviewer for this comment, we have revised any remaining terminology to adhere to recommendations chartered in the 2024 UNAIDS Terminology Guidelines. These revisions have been applied consistently throughout the main text, figure legends, tables, and supplementary materials.

2) Figure quality and consistency
   a. Several figures are of poor graphical quality, making them difficult to interpret.
   b. Figure legends are inconsistent across the manuscript. For example, healthy controls are referred to as "HC" in some figures and "Healthy" in others. This must be standardized.
   c. Color coding is also inconsistent. In Figure 3, the same groups are represented by different colors across subpanels, which is confusing and unacceptable.

   Response: We thank the reviewer for pointing out issues on figure quality and consistency. We have carefully revised all figures and legends to address these concerns.
   (a) We have ensured that high resolution figures with >300 dpi are submitted along with the revised manuscript to ensure clarity and readability in print and digital formats.
   (B) We have standardized the terminology across all figure legends and panels. Specifically, "Healthy controls" is now used consistently throughout the revised manuscript instead of "HC". All group labels have also been harmonized accordingly.
   (C) We have corrected the color scheme in **Fig. 3** of the revised manuscript for improved interpretability and visual coherence.

3) Methodological clarity
   a. It is unclear whether the proteomic values obtained from the two analytical platforms represent absolute concentrations or relative expression levels.
   b. The methodology for Gene Ontology analysis is poorly described. It is not clear whether this analysis was performed directly through IPA or using an independent pipeline. This must be clarified.

   Response: We apologize for the confusion and have revised the manuscript for methodological clarity.

(a) The proteomic measurements represent absolute concentrations (pg/ml). We have clarified this in the Methods section and updated all relevant figure axes and legends to explicitly indicate the units in the revised manuscript.
(b) The GO and pathway enrichment analyses were performed using Qiagen IPA. We have clarified in the **Methods section** that GO annotations and related pathway analyses were conducted within this platform and through an independent pipeline.

Major concerns regarding the analysis and interpretation:

4) While groups are matched for age and compared for ethnicity and STI status at blood draw, several critical confounding factors relevant to inflammatory and immunoregulatory proteomics are not addressed. In particular, obesity and smoking status are not included or discussed. Additionally, other exclusion criteria appear to rely on physician discretion but are not clearly specified in the Methods.

   Response: We attempted to recruit to our ATN study cohort, youth and young adults who were most at risk of acquiring HIV or who were effectively living with HIV, either with a recent diagnosis or with a longer duration of infection. The demographics of our study population highly mirrored that of the general population of youth living with or at risk for HIV in the United States.[25] (DeMonte, reference in the manuscript). We have performed stratified analyses in response to the prior reviewer's requests regarding STI prevalence to remove potential cofounders related to concurrent infections. Nevertheless, it is important to highlight that the young population of individuals with recent infection or at risk for HIV tend to have very pronounced immune activation markers. We did not discuss our findings within the context of body mass index or tobacco, alcohol or drug exposure as we did not want to over stratify the cohort, considering that we have a limited sample size of 262 participants, all of whom were recruited from the community or community clinics and are therefore representative of the populations of youth at risk for HIV in Los Angeles and New Orleans. We believe our data provide important information regarding immune activation markers in youth living with HIV.

   The exclusion criteria for study participation as detailed in the methods was geared towards enabling follow-up of individuals in a cohort study. Individuals who were unable to attend clinic visits and consent to study participation because of altered mental status due to active drug or alcohol use could not be enrolled as per IRB guidelines, since they were unable to provide informed consent. This assessment was performed by the site investigator, and this is what is meant by assessment of participation at the discretion of the site investigator, since only the person on site could evaluate the mental status of potential participants. Due to space limitations, we could not detail the study protocols, but they are extensively referenced in the methods section.[22-24, 54]

5) (a) In Figure 1B, the tight clustering of NY samples at 24 months is unexpected. One would anticipate these samples to move closer to healthy controls over time. Instead, they form a distinct cluster, raising concerns about potential technical bias. Were samples randomized during processing? Was batch effect assessed and corrected?
   (b) Furthermore, the description of Figure 1B in the text is overstated. The claim that NY 0M samples are more separated from HC than NY 12M is not convincingly supported by the PCA. Differences in ellipse size are subtle and do not justify the strong biological interpretation provided.

   Response: We thank the reviewer for the thoughtful comments.
   (a) We sincerely apologize for the error in the PCA plot shown in Fig. 1B. Upon re-evaluation, we have corrected the PCA plot in the revised manuscript and we also reviewed all other PCA plots to ensure accuracy. The updated analysis is now reflected in the **new Fig. 1B** of the revised manuscript.

Samples were randomized during processing, and appropriate controls were included in each batch to monitor and account for potential batch effects. We did not observe clear evidence of batch-driven clustering in the PCA analysis.
(b) We have revised the text in the **Results** section to provide accurate and more balanced interpretation. We have also toned down the description of **Fig. 1B** in the main text as reflected in the revised manuscript.

6) In Figure 2C, it is unclear which group (HC or HRY) shows upregulation or downregulation of proteins. This information should be explicitly annotated in the figure. Adjusting the log2 fold-change scale would also substantially improve readability.

   Response: The volcano plot demonstrates proteins that are upregulated and downregulated in the HRY group relative to healthy controls as indicated in the original figure legend. To further improve clarity and readability, we have now added the heading "HRY (relative to healthy controls)" directly to **Fig. 2C** and adjusted the log2 fold-change x-axis scale. Similarly, we applied the same revisions to **Fig. 4C** by adding the corresponding heading ("PY (relative to healthy controls)") and adjusting the x-axis scale in the revised manuscript to better visualize the differentially regulated proteins in the PY group relative to healthy controls.

7) In Figure 2F, it is unclear why only upregulated proteins were included in the network analysis. Downregulated proteins may be equally informative and should not be excluded without justification. Moreover, it is not clear whether the proteins shown correspond to those in Figure 2C, as the nomenclature differs.

   The figure is difficult to interpret, and the accompanying text is vague and overstated. The sentence describing a "fragmented circuit topology" is unclear and unsupported. The authors should reconsider both the figure design and the biological interpretation. What exactly is meant by "fragmented circuit topology," and how is this objectively defined?

   Response: We thank the reviewer for these critiques and apologize for the confusion. We have now revised **Fig. 2F** and the corresponding text to improve clarity and rigor.

   Figure 2f now includes all significantly dysregulated proteins. The updated analyses use z-scores to objectively predict both the activation (e.g., NF-κB, STAT3) and inhibition (e.g. IL-33 signaling) of canonical pathways, providing a balanced view of the immune landscape.

   We have now standardized the nomenclature across Figs. 2C and 2F, and clarified their relationship in the figure legend and text to ensure the proteins driving the pathway enrichments are easily identifiable.

   Finally, we have removed the term "fragmented circuit topology. The revised text in the **Results** section now describes a skewed immune profile characterized by the activation of pro-inflammatory cascades alongside the suppression of homeostatic regulators. This more accurately reflects the data, suggesting an immune environment in high-risk youth that mimics a sustained antimicrobial response.

8) In Figure 3A, the authors claim that NY 0M is the most dysregulated group and that protein expression progressively normalizes at 12 and 24 months of ART. This conclusion is not supported by the data

shown, as the three timepoints appear highly similar. A hierarchical clustering analysis of samples, colored by timepoint, would be far more appropriate to assess whether timepoints cluster separately or are intermixed. Such an analysis could reveal a persistent HIV-associated proteomic signature despite prolonged ART, which would be an important finding.

Response: We thank the reviewer for the thoughtful comment. Indeed, as shown in the Venn diagram analysis in **Fig. 3D**, there were 27 proteins (23 up- and 4 down-regulated) across all timepoints of the NY group, among which 41 proteins were significantly altered in the NY group. Additionally, we performed hierarchical clustering as suggested by the reviewer, which demonstrates an intermixed pattern, particularly among the three time points of the NY group, as shown in the **new Supplementary Fig. 2** of the revised manuscript. Hence, we have now revised the description of the results for **Fig. 3A** in the manuscript to reflect the findings in the **Results** section.

9) In Figure 3C, the authors state that PCA shows persistent separation from HC at both 12M and 24M, indicating incomplete immune reconstitution. However, 24M appears to be the most distinct group, not intermediate. This again raises concerns similar to those mentioned in my second comment.

Response: We thank the reviewer for the thoughtful comment. As mentioned above, we have added a **new Supplementary Fig. 2** and modified the description of the results for **Fig. 3A** in the manuscript to reflect accurately the findings in the **Results** section.

10) Figure 3E further strengthens concerns about potential technical or analytical bias. Pathways are consistently upregulated at 24M, regardless of whether they are also enriched at 0M or 12M. This pattern is biologically puzzling. If these pathways reflect acute infection–related inflammation, one would expect higher activation at 0M with normalization over time. Conversely, if they represent chronic inflammation under ART, one would expect low levels at 0M and progressive increases. The consistent maximal upregulation at 24M lacks interpretation and is not adequately discussed.

Response: We thank the reviewer for this critical evaluation of the longitudinal trends. However, we would like to reiterate that all samples (0M, 12M, and 24M) were randomized across processing batches, appropriate controls were included in each batch for normalization, and robust batch-correction methods were applied to monitor and account for potential batch effects. Re-evaluation of the data (**revised Fig. 3c**) confirmed that batch-driven clustering is driven by biological time points rather than processing batches.

The pathway analysis was conducted using Qiagen Canonical Pathway Analysis. The activation z-score is used to predict pathway activation (≥2) or inhibition (≤−2) by comparing observed molecular changes with literature-based expectations, inferring the direction of pathway activity based on known causal relationships, and the input expression data. Importantly, this approach does not quantify the magnitude of pathway activation but rather estimates the likelihood of a pathway being activated or inhibited in the NY group. Accordingly, our analysis is intended to provide insights into the potential immune status of NY individuals across time points, rather than to definitively establish which specific pathways are upregulated or downregulated at each time point. We have clarified this and now expanded the description of **Ingenuity Pathway Analysis (IPA)** in the **Methods** section of the revised manuscript for better clarity on the analysis performed.

Finally, we agree that the 24M pattern in **Fig. 3e** requires a precise description. We have revised the **Results** section to move away from an “acute” interpretation, instead describing a non-resolving, chronic inflammatory state that persists or stabilizes despite viral suppression.

11) In Figure 3F, it is unclear how upstream regulators were inferred. They appear to be proteins from the original dataset rather than results of a distinct upstream regulator analysis. This needs clarification. Including HC as a reference group would also strengthen this analysis.

Response: In Fig. 3F, upstream regulators that are consistently upregulated across all time points in NY individuals are presented. This analysis was conducted using Qiagen IPA software, which predicts upstream regulatory molecules that may drive the observed gene expression changes; these predictions are not necessarily derived directly from the proteomics data. The analysis was based on fold-change values calculated relative to healthy controls, which were therefore already incorporated as the reference group. We have now expanded the description of **Ingenuity Pathway Analysis (IPA)** in the **Methods** section of the revised manuscript for better clarity on the analysis performed.

12) In Figure 3G, p-values are missing and should be provided.

Response: We apologize for the omission of the *p*-values in Figure 3G, which must have been due to an image file conversion error during upload to the author submission portal in the initial submission. We have now corrected this issue and ensured that all statistical significance annotations are clearly displayed in the revised figures.

13) In Figure 4C, as in Figure 2C, the direction of regulation (which group shows upregulation or downregulation) should be clearly indicated to facilitate interpretation.

Response: The volcano plot depicts proteins that are upregulated and downregulated in each study group relative to healthy controls, as indicated in the original figure legend. To further improve the readability and clarity, we have adjusted the x-axis scales and added explicit headings directly to the figures: "HRY (relative to healthy controls)" for **Fig. 2C** and "PY (relative to healthy controls)" for **Fig. 4C** in the revised manuscript.

14) In line 221, TNF is incorrectly described as an immunosuppressive mediator, whereas it is a well-established pro-inflammatory cytokine.

Response: This statement has been corrected to indicate TNF's pro-inflammatory profile.

15) The identification of 10 proteins downregulated in the HRY group is one of the most interesting findings in the manuscript, yet it is underdeveloped. These proteins are largely pro-inflammatory. This raises an important and intriguing hypothesis: could a lower inflammatory baseline contribute to protection from HIV acquisition? This aspect of the HRY group is insufficiently discussed and represents a missed opportunity.

Response: We thank the reviewer for this insightful observation regarding the 10 downregulated proteins in the HRY group. We agree that this disrupted inflammatory profile, where specific pro-inflammatory mediators are suppressed while others are heightened, is a pivotal finding that warrants deeper discussion. We have expanded our **Discussion** of this phenomenon and now hypothesize a dysfunctional innate alerting system, given that many of these downregulated proteins are involved in early pathogen recognition and leukocyte recruitment. Thus, their suppression may suggest a "blunted" initial response to viral exposure, potentially influencing susceptibility to infections. Additionally, the concurrent upregulation of NF-κB and STAT3 pathways alongside the downregulation of these 10 proteins suggests that HRY individuals are "maladaptively primed". However, the relationship between immune activation and HIV acquisition is complex and warrants further investigation. We have integrated and discussed these points in the **Discussion** section of the revised manuscript to emphasize that this specific proteomic signature may be a marker of

increased vulnerability rather than protection, potentially explaining why these individuals remain at high risk despite the absence of a uniform inflammatory profile.

16) Figures 5C and 5D are of very poor graphical quality and use different color palettes. These figures should be reformatted for clarity and consistency.

Response: We apologize for the poor graphical quality in the initial submission, which appears to be due to an image compression error during the portal's conversion process during the first submission. We have now provided high-resolution versions of **Figs. 5C** and **5D** to reflect the same consistent color palette in the revised manuscript. The figure legends and corresponding results in the main text have also been revised to accurately match what was presented in the figure.

17) In Figures 5E–F, the discussion in the text is minimal and confusing. CXCL10 is mentioned in the text, but the figure displays CXCL9. Similar inconsistencies occur elsewhere. Additionally, CCL2 is discussed but not visible in the figure.

Response: We sincerely apologize for the oversight and thank the reviewer for the careful assessment. We have performed a comprehensive check of the **Results** section to ensure complete alignment between the text and the figures.

We have corrected the main text description to accurately reflect the results as shown in **Figs. 5E-F** of the revised manuscript. Moreover, we have standardized the nomenclatures for the remaining figures for visual consistency for the reader. More importantly, we have expanded our **Results** and **Discussion,** ensuring that the proteins presented in the figures were mentioned and properly discussed in the revised manuscript.

18) In summary, while the cohorts are valuable and the study has the potential to make a meaningful contribution to the field, the manuscript in its current form is not ready for publication. Substantial revisions are required, including improvements in writing, methodological transparency, figure quality, statistical rigor, and biological interpretation. Without these changes, the data remains underutilized, and many conclusions are insufficiently supported.

Response: We thank the reviewer for the comprehensive and critical assessment of our manuscript. We recognize that the concerns raised reflect critical issues regarding the overall rigor, clarity, and interpretability of the study, and we have undertaken extensive revisions to address each of these points in a systematic manner.

First, we have substantially revised the writing throughout the manuscript to improve clarity, precision, and consistency, including the adoption of standardized, person-first terminology and removal of ambiguous or overstated language.

Second, we have strengthened methodological transparency by expanding the Methods section to clearly describe proteomic quantification, data pre-processing, batch control procedures, and pathway analysis workflows.

Third, all figures have been carefully reworked to improve quality and interpretability. This includes replacement with high-resolution images, consistent labeling and group nomenclature, standardized color schemes across panels, and clearer annotation of directionality in differential expression analyses, and correction of previously identified errors.

Fourth, we have improved the analytical rigor of the study by incorporating additional analyses in response to reviewer comments. These include stratified analyses to address potential confounders (e.g., STI status), as well as hierarchical clustering to more appropriately assess longitudinal proteomic patterns.

Finally, we have revised the **Results and Discussion** extensively to ensure that all interpretations are directly supported by the data. Specifically, we have moderated overstatements, clarified ambiguous descriptions, and provided more precise biological context for key findings.

Taken together, these revisions represent substantial changes across all major aspects highlighted by the reviewer, which we are grateful for. We believe that the manuscript has been significantly strengthened and now provides a more rigorous, transparent, and data-supported account of the findings.

Response to Reviewers: NCOMMS-25-97667-T

Reviewer #1:

The authors have adequately addressed my previous concerns, and the revised manuscript has significantly improved. I have no further comments.

Response: We sincerely thank the reviewer for their time, careful evaluation, and positive assessment of our revised manuscript. We are deeply grateful for the constructive feedback provided throughout this process, which has significantly enhanced the clarity, rigor, and overall quality of our work.

Reviewer #2:

The authors have clearly engaged with my prior comments and made several constructive improvements (including figure corrections/updates and additional stratified analyses), which I appreciate. However, despite these revisions, the overall conclusions remain unconvincing because key analytical steps and quality-control measures are still insufficiently described, and the manuscript continues to draw mechanistic inferences that are not adequately supported by the presented data. The central claims (e.g., "blunted innate sensing," "suppressed type I IFN signaling," "NF-κB/STAT3 activation," "immune exhaustion/priming," cGAS–STING involvement) remain unsupported by orthogonal cellular or functional data as they are based on inference from measurement of 49 plasma proteins. As written, the study is best framed as descriptive proteomic profiling, not mechanistic immunology.

Response: We thank the reviewer for this conceptually rigorous and essential critique, which has significantly enhanced the clarity, rigor, and overall quality of our work. As the design for our clinical cohort was limited to the collection of plasma samples, matching peripheral blood mononuclear cells (PBMCs) were not available for orthogonal cellular and functional assay validation to confirm the involvement of predicted pathways. To ensure that our conclusions accurately reflect the boundaries of our data and align with our reviewer's excellent recommendation, we have comprehensively reframed the manuscript to focus on descriptive proteomic profiling and predictive pathway analyses for hypothesis-generation. Specifically, we: (i) systematically reframed the Ingenuity Pathway Analysis (IPA) outputs explicitly as predictive, with a hypothesis-generating insight rather than definitive mechanistic proof; (ii) expanded the ***Limitations and Future Directions*** section to explicitly outline the boundaries of peripheral proteomic data and the necessity of future functional verification; and (iii) revised our terminology throughout the manuscript text, replacing definitive assertions regarding upstream signaling states with predictive or associative language. We believe these extensive revisions successfully harmonize our claims with the descriptive nature of the data, and we thank the reviewer for elevating the precision of our study.

Major comments:

1) Data processing and normalization.
The Methods state that proteomic data were "log-transformed and normalized prior to analysis," and that PCA was used to visualize global patterns. However, the manuscript does not specify what normalization approach was applied (e.g., median/quantile/plate-wise, internal standards, bridging controls), whether batch effects were formally evaluated, or whether any batch correction was applied (and if so, using which method and covariates). Given that PCA/clustering are central to the narrative, and the authors corrected an error in the PCA figure,

it is important to demonstrate that the observed structure is not driven by processing batches, plate effects, site, or run date.

Please provide:
a) a clear description of normalization and any batch correction method used
b) evidence that batch effects were assessed (e.g., PCA colored before and after correction)

Response: We thank the reviewer for this critical comment which has enabled us to improve the clarity and rigor of our study in this revision.

a) We appreciate the reviewer's comment. A formal batch effect analysis was not performed because the Luminex assays were conducted under standardized conditions with internal quality control measures. Assay quality was monitored using calibration standards, internal controls, duplicate measurements, bead counts, standard curve performance, and intra-assay CVs. These quality control procedures demonstrated consistent assay performance across all analyzed samples. As each Luminex plate could only accommodate 38 to 40 samples, the entire batch of 262 specimens were analyzed across multiple plates. To minimize plate-to-plate variation, three bridging controls were included on every Luminex plate. The bridging controls enabled accurate normalization across plates and ensured consistency of protein measurements throughout the entire dataset. We have now included these descriptions in the ***Methods*** section, under the ***Plasma Proteomics Profiling*** section of the revised manuscript.
b) We appreciate the reviewer's comment. Over the course of revisions, we have standardized all PCA plots to be generated using GraphPad Prism to ensure consistency throughout the manuscript. As described in our response to the above comment (a), appropriate internal quality control measures, including calibration standards, internal controls, and bridging control samples for inter-plate normalization, were implemented during the Luminex assays. Therefore, no additional formal batch effect assessment or correction was performed. To further validate the PCA generated using GraphPad Prism, we independently re-analyzed the dataset presented in **Fig. 1B** using R (v4.3). The PCA generated in R produced an identical spatial distribution of samples and identical variance calculations (PC1=31.75%, PC2=10.86%), confirming the accuracy, robustness and reproducibility of the PCA analyses presented in the manuscript (**Fig. R1**).

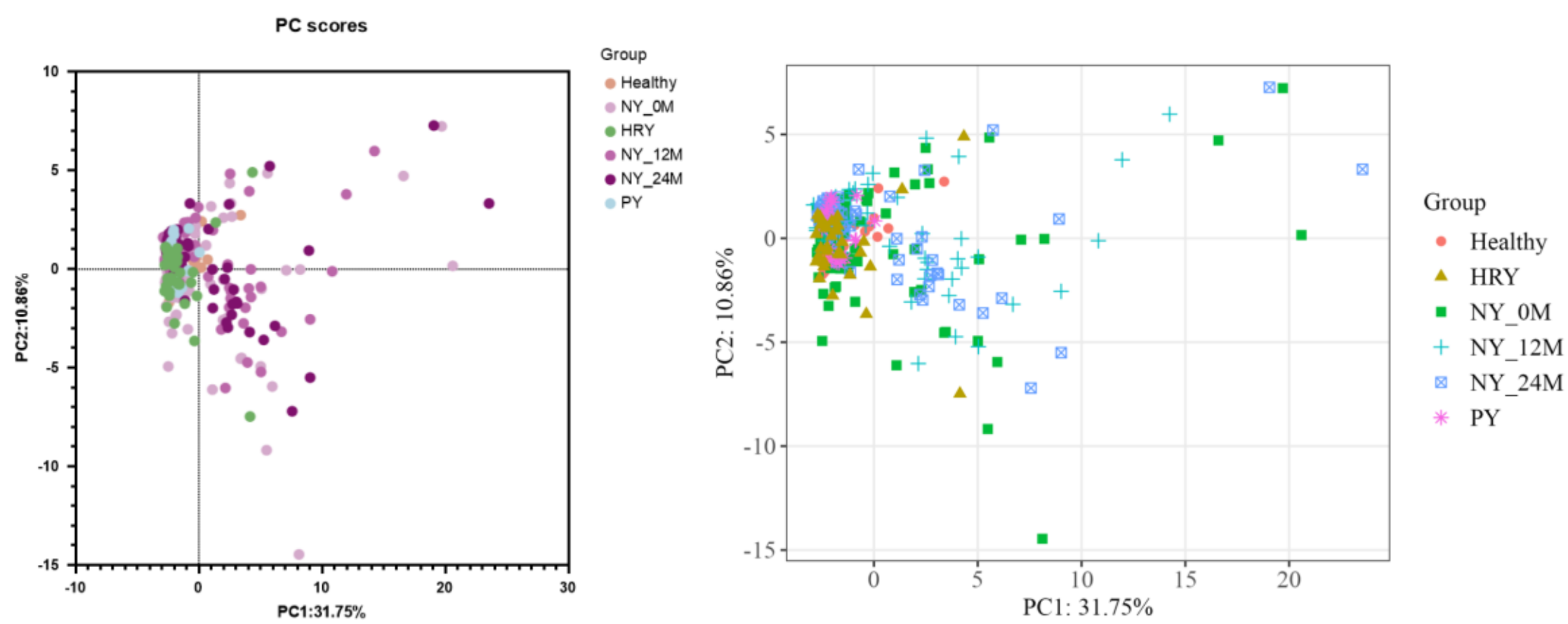

Figure R1. **Cross-platform validation of the PCA framework for Figure 1B.** The spatial distribution, point topology, and calculated variance for PC1 (31.75%) and PC2 (10.86%) are identical between GraphPad Prism (left) and R (right).

2) Mechanistic claims from IPA based on plasma proteins are overreaching.

The manuscript continues to infer “upstream regulator activation/inhibition” (e.g., NF-κB, STAT3, type I interferon) and pathway status (e.g., cGAS–STING) from directional changes in a limited circulating protein panel using Ingenuity Pathway Analysis (IPA). This is a methodologically precarious leap. Plasma cytokine/chemokine concentrations reflect a net outcome of secretion, compartmentalization, receptor shedding, clearance kinetics, binding proteins, and consumption and not intracellular transcription factor activity. Consequently, claims of “activation” or “inhibition” of master regulators based on extracellular concentrations alone are over-interpretations. What orthogonal evidence supports the predicted upstream regulator states, beyond IPA extrapolation from 49 circulating proteins? I would suggest also some mechanistical validations to strength the hypothesis and observations, such as: (a) PBMC transcriptomics (bulk or single-cell) or targeted qPCR (to check for IFN stimulated genes, NF-kB, etc) (b) intracellular signaling readouts (phospho-flow / CyTOF), e.g., pSTAT1/2 (type I IFN), pSTAT3, p-p65 (NF-κB).

Response: We thank the reviewer for the insightful feedback. We agree with the reviewer that this study is best characterized as descriptive plasma proteomic profiling rather than mechanistic immunology. We apologize for the overinterpretation of the IPA results in the original manuscript.

In response to the reviewer’s comments, we have comprehensively revised the manuscript to present all IPA outputs as exploratory, predictive and hypothesis-generating, rather than evidence of definitive mechanistic changes. We have also refined our biological interpretations of plasma proteomic data. We have refined our biological interpretations to ensure they remain appropriately grounded within the scope of circulating plasma proteomics. Throughout the revised manuscript, we have removed language implying direct measurement or demonstration of intracellular signaling activity, causal mechanisms, or pathway activation states. Consequently, our conclusions are now carefully framed to reflect the descriptive nature of the proteomic data while highlighting biologically plausible hypotheses that warrant further mechanistic investigation.

As noted in our earlier response, the clinical cohort was designed to collect plasma specimens only. Therefore, matched PBMCs were not available for orthogonal cellular or functional validation of the predicted pathways. While we acknowledge this as a limitation of the current study, we do not believe it diminishes the biological and clinical insights provided by the plasma proteomic profiling. Rather, these findings establish a valuable framework for future studies incorporating matched cellular samples and functional assays to validate the predicted immune pathways and elucidate their mechanistic roles in disease pathogenesis. We have included these limitations in the ***Conclusion*** section, under the ***Limitations and Future Directions.***

3) “Immune exhaustion” and “immune priming”.
The manuscript links elevated soluble checkpoint-related markers (notably soluble PD-L1) to “immune exhaustion,” and interprets the HRY profile as “primed/maladaptively primed.” However, no functional assays are presented to support exhaustion, dysfunction, or altered responsiveness.
Soluble checkpoint molecules are not sufficient proxies for T cell exhaustion. Exhaustion is a cell-state defined by single-cell phenotype and function (co-inhibitory receptor co-expression, transcriptional state, impaired proliferation/cytokine production, etc.), not by plasma marker levels alone. They should replace “exhaustion” language with conservative phrasing (e.g., “increased soluble immune checkpoint markers” or “checkpoint-associated signature”) unless functional data are added. Clearly distinguish correlation from mechanism throughout the Discussion.

Similarly, functional data would be required to demonstrate real exhaustion of HRY Tcells: proliferation assay (CFSE/CTV), cytokine production after stimulation (anti-CD3/CD28; PMA/ionomycin) and cytotoxic markers along with a flow characterization of exhaustion markers (PD-1, TIGIT, LAG-3, TIM-3, CD39, TOX) plus memory/activation context.

Response: We thank the reviewer for this crucial conceptual critique and thoughtful suggestion to emphasize the correlative nature of our findings throughout the ***Discussion***. We apologize for the overinterpretation of the plasma proteomics data in the original manuscript and acknowledge that the lack of matched PBMCs precluded orthogonal cellular or functional validation of the predicted pathways. We have comprehensively revised the manuscript to appropriately align our conclusions with the descriptive, exploratory nature of plasma proteomics profiling. While we acknowledge this as a limitation of the current study, we do not believe it diminishes the biological and clinical insights provided by the plasma proteomic profiling. Rather, these findings establish a valuable framework for future studies incorporating matched cellular samples and functional assays to validate the predicted immune pathways and elucidate their mechanistic roles in disease pathogenesis. We have included these limitations in the ***Conclusion*** section, under the ***Limitations and Future Directions.***

4) STI confounding: stratification helps but is still not fully convincing for the strongest claims The authors added STI-stratified analyses (response document pp. 1–2), concluding STI status "did not significantly alter general immune profiles." This is helpful, but the interpretation remains too definitive.

For the key conclusions (e.g., "blunted IFN," "primed state"), re-run and show results restricted to STI-negative participants only and indicate whether the same IPA calls persist with comparable effect sizes. Do the signature proteins and IPA predictions remain when analyses are strictly restricted to STI-negative participants, and do they survive correction with adequate power?

Response: We thank the reviewer for this regarding the potential confounding influence of STI status on our analyses. To directly address the reviewer's concern, we performed additional comparative analyses stratified by STI status and generated ***new Figs. S2 and S4***, with the corresponding ***Results*** incorporated into the revised manuscript. Specifically, we performed the following analyses:

a. ***Global proteomic variance and sample topology (PCA):*** As shown in **new Figs. S2A-B** (HRY) and **Fig. S4** (NY) group across longitudinal timepoints, new PCA was performed after stratifying participants according to STI status. Across both cohorts, STI(-) and STI(+) participants exhibited substantial overlap, with no distinct clustering based on STI status. These findings indicate that STI status does not drive the dominant global variance of the proteomic profiles in either cohort, validating our prior findings.
b. ***Conservation of core proteomics signatures (DEP overlap analysis):*** To further confirm whether the identified immune signatures were preserved when analyses were restricted to STI(-) participants, we compared differentially expressed proteins (DEPs) identified in STI(-) and STI(+) groups relative to Healthy controls. As illustrated in the Venn diagrams (**Figs. S2B, S4D-F**), apart from largely overlapped profiles in STI(-) and STI(+) groups, both groups demonstrate minimal unique DEPs across the HRY and NY (timepoints) comparisons. Given the minimal number of unique DEPs, independent IPA of the subgroup-specific proteins would not be informative or statistically robust. Collectively, these analyses demonstrate that the core plasma proteomic signatures identified in this study are highly conserved irrespective of STI status, supporting the robustness of our findings.

Minor / specific comments:

1. Figure 1B: The spread/dispersion of groups should be discussed with respect to cohort heterogeneity. Could the wider dispersion in some groups reflect unequal STI burden or other measured covariates? Please explicitly test whether dispersion correlates with STI status or other available metadata.

Response: We thank the reviewer for this insightful question and suggestion. As suggested, we performed a two-step statistical verification to evaluate the effects of STI status and main cohort variables on multivariate dispersion and have expanded the ***Results*** and ***Discussion*** sections to address this cohort heterogeneity of the revised manuscript. Specifically, we have performed:

a. ***Assessment of STI status:*** As demonstrated in the **new Fig. S2** (and detailed in our response to Major comment 4), stratified PCA revealed near-identical spatial distribution and dispersion patterns between STI(+) and STI(-) participants across all groups and timepoints, indicating that STI burden does not drive the observed sample dispersion.
b. ***Formal Dispersion analysis (PERMDISP):*** To evaluate the homogeneity of multivariate dispersion among groups, we performed a permutational analysis of multivariate dispersions (PERMDISP) using the vegan package in R (PMID: 16542252). An Euclidean distance matrix was first calculated using the vegdist() function followed by measurement of the distance to centroid, which measures dispersion within each group, calculated with the betadisp() function. As the distance metrics were normally distributed, group differences were assessed using a one-way Welch's ANOVA followed by post-hoc Dunnett's T3 multiple-comparison test to compare groups relative to healthy controls. As shown in the **new Fig. S1**, PERMDISP showed a significant difference in dispersion (P=0.0003) across the study groups. Post-hoc analysis demonstrated that this variation is heavily driven by unequal dispersion between NY at 0 months (0M) compared with healthy controls (P=0.0006), together with significant longitudinal changes following ART initiation (0M vs 12M, P=0.0018; 0M vs 24M, P=0.0076). Conversely, within-group dispersion was highly homogeneous and statistically distinguishable when comparing healthy controls against the PY or HRY cohorts.

2. Figure 2 (HRY profiling): Some upregulated markers are in the β-chemokine family (e.g., CCR5 ligands) which can competitively inhibit HIV entry. This alternative biological interpretation (potential protective/competitive mechanism rather than "blunted sensing") should be discussed.

Response: We thank the reviewer for this insightful alternative biological interpretation. The upregulated pro-inflammatory mediators we identified in the HRY cohort included β-chemokines, specifically CCL3 and CCL4 which are natural ligands for the CCR5 co-receptor. When elevated systemically, they can competitively inhibit HIV-1 gp120 binding, thus acting as a potential protective barrier against viral entry rather than solely representing a "blunted sensing". We completely agree that this dual nature, where a profile appears to be globally dysregulated yet contains specific components that could prevent viral fusion, adds vital immunological depth to our interpretation of the HRY cohort. We have revised our ***Results*** and ***Discussion*** sections to explicitly highlight this potential protective, competitive entry mechanism alongside our original observations for a balanced interpretation of our findings.

3. Terminology mismatch (HRY vs HIV infection): In the description of Fig. 2E/related text, the phrase "inflammatory network in HIV infection" is inaccurate if referring to HRY who are HIV-negative. Please correct to avoid conceptual confusion.

Response: We sincerely apologize for this oversight and thank the reviewer for identifying this critical typographical error. We have corrected this phase in the ***Results*** section to accurately refer to this high-risk cohort.

4. IPA general limitation: Please add explicit limitation language: IPA upstream regulator analysis is not direct evidence of intracellular activation/inhibition, especially when input is limited to circulating proteins.

Response: We agree with the reviewer's recommendation and have explicitly integrated this parameter into our ***Limitations and Future directions*** section, stating directly that IPA upstream regulator analysis extrapolated from a circulating protein panel provide a descriptive footprint and does not constitute direct evidence of intracellular activation or inhibition.

5. No validation/perturbation: The manuscript should not read as if mechanisms were demonstrated. If the authors cannot add functional experiments, the Discussion should be rewritten to clearly present hypotheses and avoid therapeutic suggestions that rely on unproven mechanistic states.

Response: We thank the reviewer for this constructive guidance. Because additional functional perturbation or *ex vivo* cellular experiments could not be performed to the plasma-only nature of this archived repository, we have thoroughly audited and rewritten the ***Discussion*** section to remove any language implying that mechanisms were definitively demonstrated. We also reframed our conclusions as exploratory, hypotheses-generating models and heavily qualified any speculative therapeutic suggestions (such as checkpoint blockade or anti-inflammatory adjunctive therapies) to ensure they are presented strictly as long-term conceptual possibilities rather than immediate clinical recommendations in the revised in ***Discussion*** and ***Limitations and Future directions*** sections.

6. Nomenclature consistency: Ensure consistent labeling between text and figures (e.g., eotaxin vs CCL11; GRO1 vs CXCL1, etc.). This inconsistency was raised before at other figures in my first review, but those specific molecules of figure 5 were correctly labeled in the previous version.

Response: We thank the reviewer for the thoughtful comment. We thoroughly reviewed the entire manuscript text, figures, tables, and figure legends to ensure absolute nomenclature consistency. Following the standards established by the International Union of Immunological Societies (IUIS) (PMID:11527998) and UniProt (PMID: 39552041), we have adopted the modern, systematic nomenclature for all immune mediators throughout the manuscript (i.e., using CCL11 instead of eotaxin and CXCL1 instead of GRO). Regarding the reviewer's comment on **Fig. 5**, we have corrected the labels to ensure they are fully aligned with the systematic nomenclature used in the text, resolving the discrepancies introduced during our recent revisions. To maintain clarity for all readers, we have explicitly redefined each immune mediator using both systematic (CD/chemokine) nomenclature and its legacy common/functional name upon its first mention in the ***Methods*** (***Plasma Proteomic Profiling*** subsection) and added the corresponding reviewed UniProt IDs for all the relevant protein markers in **Table 2**..

7. Psychosocial stressors: The Discussion mentions psychosocial stressors as drivers (your noted lines ~314–315). If authors invoke this, they should either (i) analyze available stress-related metadata, or (ii) clearly label this as speculation and expand the confounding

discussion. [Gregor MF, Hotamisligil GS. Inflammatory mechanisms in obesity. Annu Rev Immunol (2011); Arnson Y, Shoenfeld Y, Amital H. Effects of tobacco smoke on immunity, inflammation and autoimmunity. J Autoimmun (2010).

Response: We thank the reviewer for this valuable point. Because standardized, quantitative psychosocial stress-related metrics were not systematically collected for these clinical cohorts, we have explicitly labeled this mechanism as speculative. Following the reviewer's recommendation, we expanded the ***Discussion*** of this unmeasured environmental confounding (***paragraph 3***). We also integrated and cited the recommended literature to illustrate how non-infectious, systemic stressors such as metabolic inflammation (e.g., obesity) or lifestyle exposures (e,g., tobacco smoke), can independently drive plasma proteomic alterations (PMIDs: 2129177; 20042314).
